# Developing a database for pedestrians’ earthquake emergency evacuation in indoor scenarios

**DOI:** 10.1371/journal.pone.0197964

**Published:** 2018-06-20

**Authors:** Junxue Zhou, Sha Li, Gaozhong Nie, Xiwei Fan, Jinxian Tan, Huayue Li, Xiaoke Pang

**Affiliations:** 1 Institute of Geology, China Earthquake Administration, Beijing, China; 2 Earthquake Administration of Guangxi Zhuang Autonomous Region, Nanning, China; 3 China Earthquake Networks Center, Beijing, China; Beihang University, CHINA

## Abstract

With the booming development of evacuation simulation software, developing an extensive database in indoor scenarios for evacuation models is imperative. In this paper, we conduct a qualitative and quantitative analysis of the collected videotapes and aim to provide a complete and unitary database of pedestrians’ earthquake emergency response behaviors in indoor scenarios, including human-environment interactions. Using the qualitative analysis method, we extract keyword groups and keywords that code the response modes of pedestrians and construct a general decision flowchart using chronological organization. Using the quantitative analysis method, we analyze data on the delay time, evacuation speed, evacuation route and emergency exit choices. Furthermore, we study the effect of classroom layout on emergency evacuation. The database for indoor scenarios provides reliable input parameters and allows the construction of real and effective constraints for use in software and mathematical models. The database can also be used to validate the accuracy of evacuation models.

## Introduction

China is an earthquake-prone country. It is also among the countries that have suffered the most from a serious earthquake. Earthquake and its secondary disasters cause serious casualties and property losses, and the social impact of earthquake disaster ranks first among the different types of natural disasters. From January 2012 to May 2017, 30 earthquakes with a magnitude greater than Ms 6.0 have occurred in mainland China (China Earthquake Networks Center: www.ceic.ac.cn/). For example, the 2014 Ms 6.4 Ludian earthquake caused 617 deaths and injured 3,143 people, and 112 people went missing (as of 3 p.m. on August 8th); the 2014 Ms 6.6 Jinggu earthquake caused 1 death and injured 323 people (as of 4 a.m. on October 8th); the 2013 Ms 6.6 Zhangxian earthquake caused 89 deaths and injured 628 people, and 5 people went missing (as of 8 p.m. on July 22nd); and the 2013 Ms 7.0 Lushan earthquake caused 196 deaths and injured 11,470 people, and 21 people went missing (as of 2 p.m. on April 24th) (China Earthquake Administration: http://www.cea.gov.cn/). Given the ongoing social and economic development and the accumulation of wealth, society is becoming more vulnerable to natural disasters, such as earthquakes. The casualties and property losses caused by earthquake are becoming increasingly serious [1]. The primary cause of earthquake-related casualties is that people fail to evacuate to safe areas before the buildings they occupy collapse. Thus, the correct and efficient earthquake emergency response behavior (EERB) is closely related to human safety and survival.

For any evacuation software, data on the EERB of occupants is urgently required [2–3]. These types of software programs could be used to simulate evacuation in outdoor [3] and indoor scenarios [4–7]. To improve the accuracy of simulation results, we require qualitative and quantitative data from a crowd in an actual earthquake emergency response. Qualitative data include the response behavior of pedestrians, man–environmental interactions [8–9] and chronological decision-making processes of organizations at different evacuation stages [10–11]. Quantitative data include delay time, evacuation speed [12], evacuation route choices [13–14] and evacuation exit choices [11]. Thus, we should construct a database of EERB. Such a database can provide reliable parameters and lead to real and effective constraints for use in software and mathematical models.

At present, three primary methods have been used to collect behavioral data for earthquake emergencies: evacuation drills, VR experiments and questionnaires. Evacuation drills consume considerable manpower and material resources. More importantly, this method cannot approximate real earthquakes. The participants do not achieve the state of panic that occurs when actual earthquakes take place [15]. VR experiment is a novel approach, but the data obtained by this method are just simulated data, not the real EERB of pedestrians after the earthquake [16–17]. The results obtained from the questionnaire method contain errors or exaggerations due to the subjective nature of human awareness. That is, when earthquakes occur, people find themselves in a dramatically changed environment, and they become highly nervous and confused. After an earthquake, respondents do not want to recall the tragic experience of the earthquake [18].

In recent years, security awareness in China has improved, and an increasing number of surveillance cameras are being installed in communities. Many surveillance videos used for security purposes record the real EERB of people during disasters. Therefore, we use the Transana and Tracker software packages to objectively analyze social surveillance videos and the emergency responses of pedestrians. We also construct a database that can provide a scientific basis for the application of evacuation simulation software. The database is composed of the following aspects.

- We have built a Keyword database (Individual characteristics, Pre-earthquake phase and Pedestrian response phase), which could be used to analyze the decision-making process of pedestrian evacuation and the pattern of evacuation behavior [19].
- Individual characteristics. Due to the differences in physiological and psychological characteristics, people of different genders tend to make different decisions and adopt different behaviors [2,19].
- Pre-earthquake phase. Pre-earthquake phase refers to the state of pedestrians before the earthquake occurs, which includes location (the position of the pedestrians), social context (the pedestrians are with whom?), and action (what are they doing when the earthquake occurs?). For example, pedestrians who are moving cannot easily feel slight shaking. In contrast, pedestrians who are still can easily feel slight shaking and take protective action. The pre-earthquake phase will have a certain impact on pedestrians’ emergency response decisions [20].
- Pedestrian response phase. People will display different self-protection behaviors with different degrees of ground shaking. Their behaviors and choices would be affected by the environment and other people around them.
- Delay time includes recognition time and response time. Recognition time is the time from feeling the ground shaking to confirming the earthquake occurrence; response time is the time from the first response to taking the first protection action. Based on the particularity of earthquake disasters and the characteristics of Chinese people, self-protection actions include evacuation or hiding oneself. Thus, delay time not only refers to the time between earthquake occurrence and evacuation but also to the time between earthquake occurrence and hiding oneself.
- Evacuation speed. Some studies have previously investigated earthquake evacuation speed [2]. In this paper, the quantitative analysis method [21] is used to study the relationship between evacuation speed and seismic intensity in a location (density: person/m^2^).
- Evacuation route choices. The physical environment of evacuation route can affect the evacuation efficiency [22–23]. In this paper, evacuation routes are internal to a single room, and the four stages of pedestrian evacuation process and the factors affecting the evacuation route are analyzed.
- Emergency exit choices. The choice of emergency exit will be connected with the evacuation route and affect the evacuation efficiency [24].

One of the innovation in this paper is the analysis of the influence of seismic intensity on pedestrian evacuation. In this study, we employ the Chinese seismic intensity scale (CSIS) to represent earthquake intensity. This intensity scale is the national standard used in the People’s Republic of China [25]. When an earthquake occurs, a large number of experts in the fields of seismology and civil and structural engineering are sent to the earthquake-stricken areas. Through large-scale field surveys and considerable effort, the intensity is determined according to the CSIS [26]. The CSIS is a scientific method and has been used by the Chinese government and research institutes in China for many years. Through long-term application at multiple earthquake sites and after practical inspection, the intensity scale has been shown to be reliable. Moreover, many scientific studies are based on this intensity scale [27–31]. In this paper, we use the CSIS as a measure of the seismic effects of the chosen earthquakes.

## Videotapes and analysis methods

The collection and organization of qualitative and quantitative data is the first step in characterizing the EERB of pedestrians for use in simulations. In this paper, collected videotapes are treated as the research object and used to construct the database of EERB. This database will be valuable in developing, evaluating and validating different types of earthquake evacuation simulators, and it can be used to verify the accuracy of model results [32].

### Videotapes

The following principles were followed to select videotapes that have recorded real earthquake events in mainland China [2]. Each videotape contains only indoor scenes, thereby allowing an effective bound connected to physical environmental elements.

The videotapes used should satisfy the following criteria:

- contain at least one person;
- include confirmed information on the time and location of the earthquake;
- record people with different personal characteristics (e.g., age and gender);
- display different scenes (e.g., classrooms, offices, and supermarkets);
- reflect different social contexts (e.g., with friends or alone);
- reflect different seismic intensities (I-IX); and
- reflect earthquakes occurring in different years (2008–2016).

The selected videotapes satisfy these criteria and are representative.

**Fig 1:**
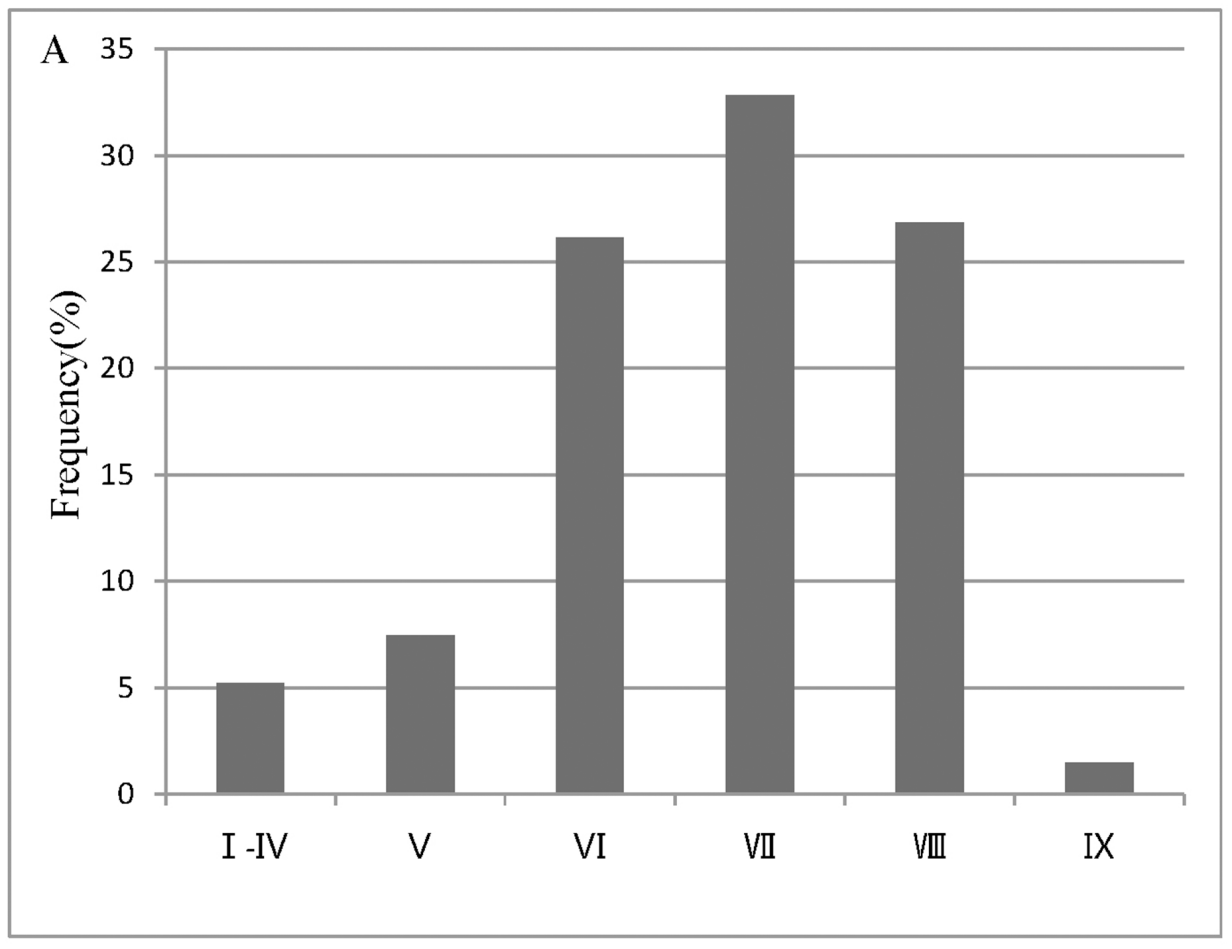
Seismic intensity according to the CSIS.

**Fig 2:**
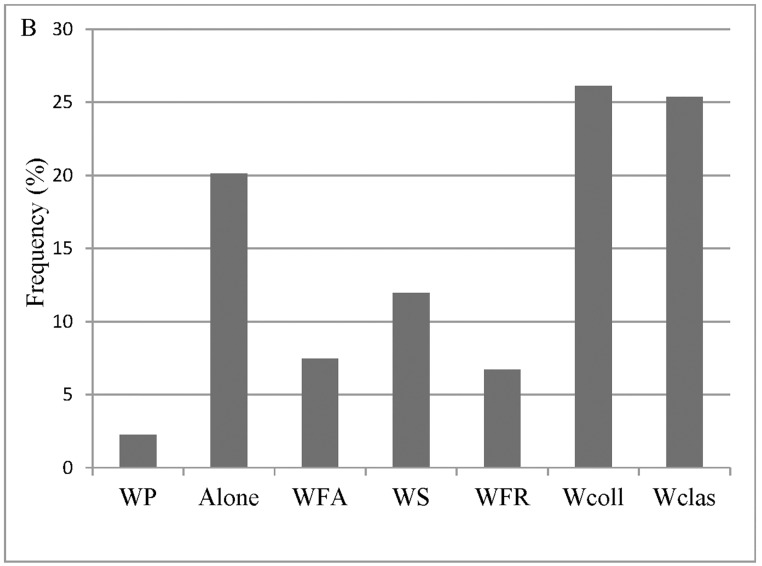
Social context. Notes: WP-With patients; WFA-With family; WS-With strangers; WFR-With friends; Wcoll-With colleagues; Wclas-With classmates.

**Fig 3:**
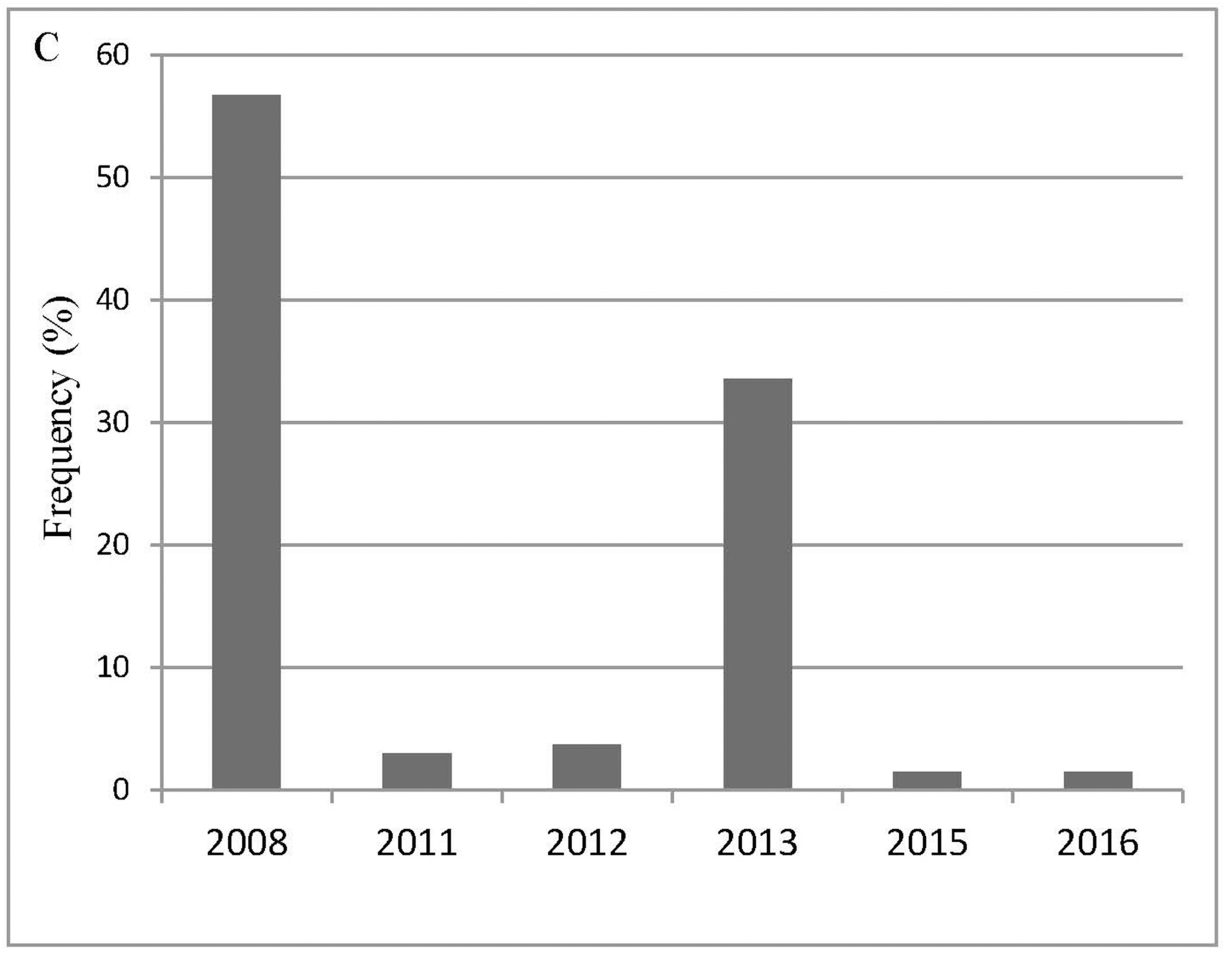
The year in which the earthquakes occurred.

**Fig 4:**
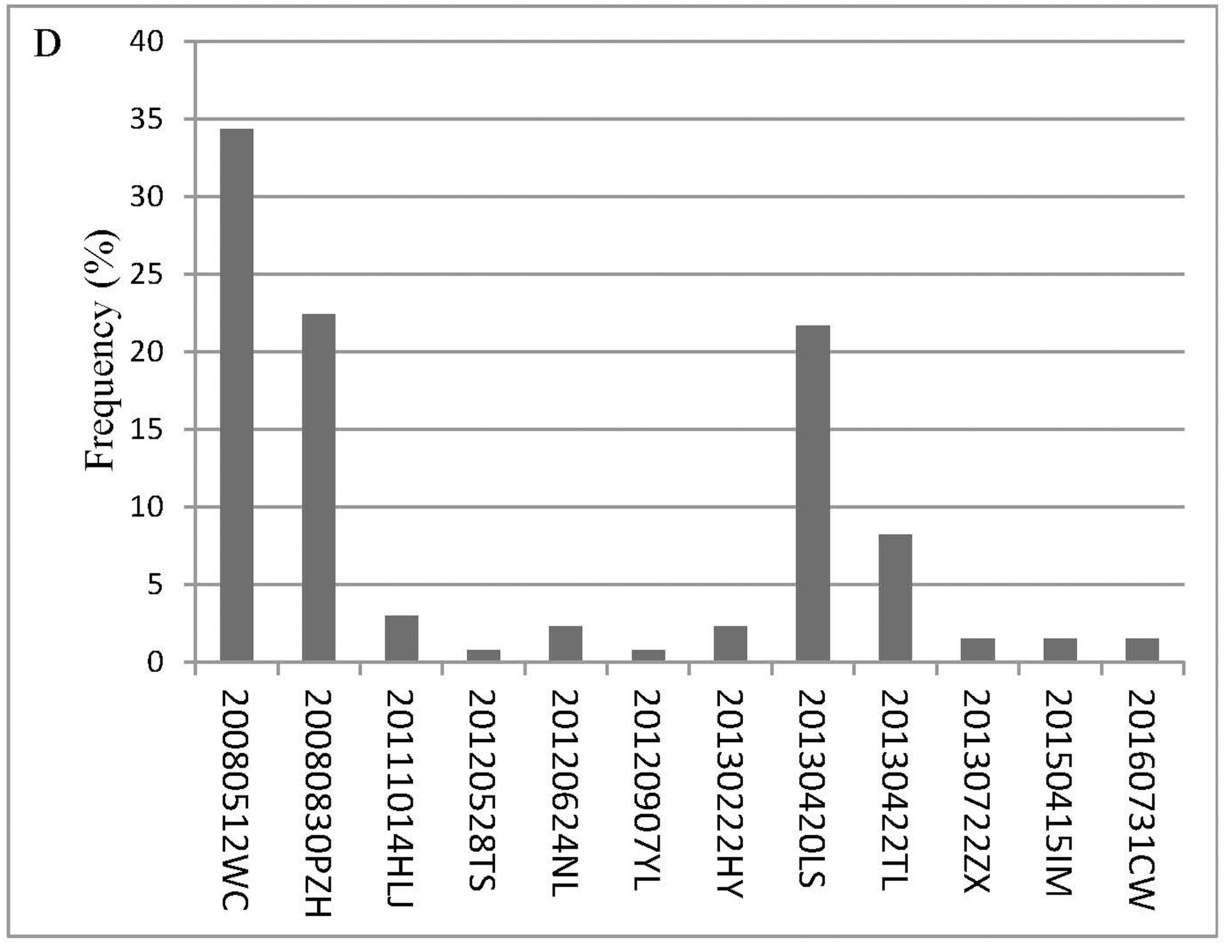
The specific earthquake. Notes: WC-Wenchuan; PZH-Panzhihua; HLJ-Heilongjiang; TS-Tangshan; NL-Ninlang; YL-Yiliang; HY-Heyuan; LS-Lushan; TL-Tongliao; ZX-Zhangxian; IM-Inner Mongolia; CW-Cangwu.

**Fig 5:**
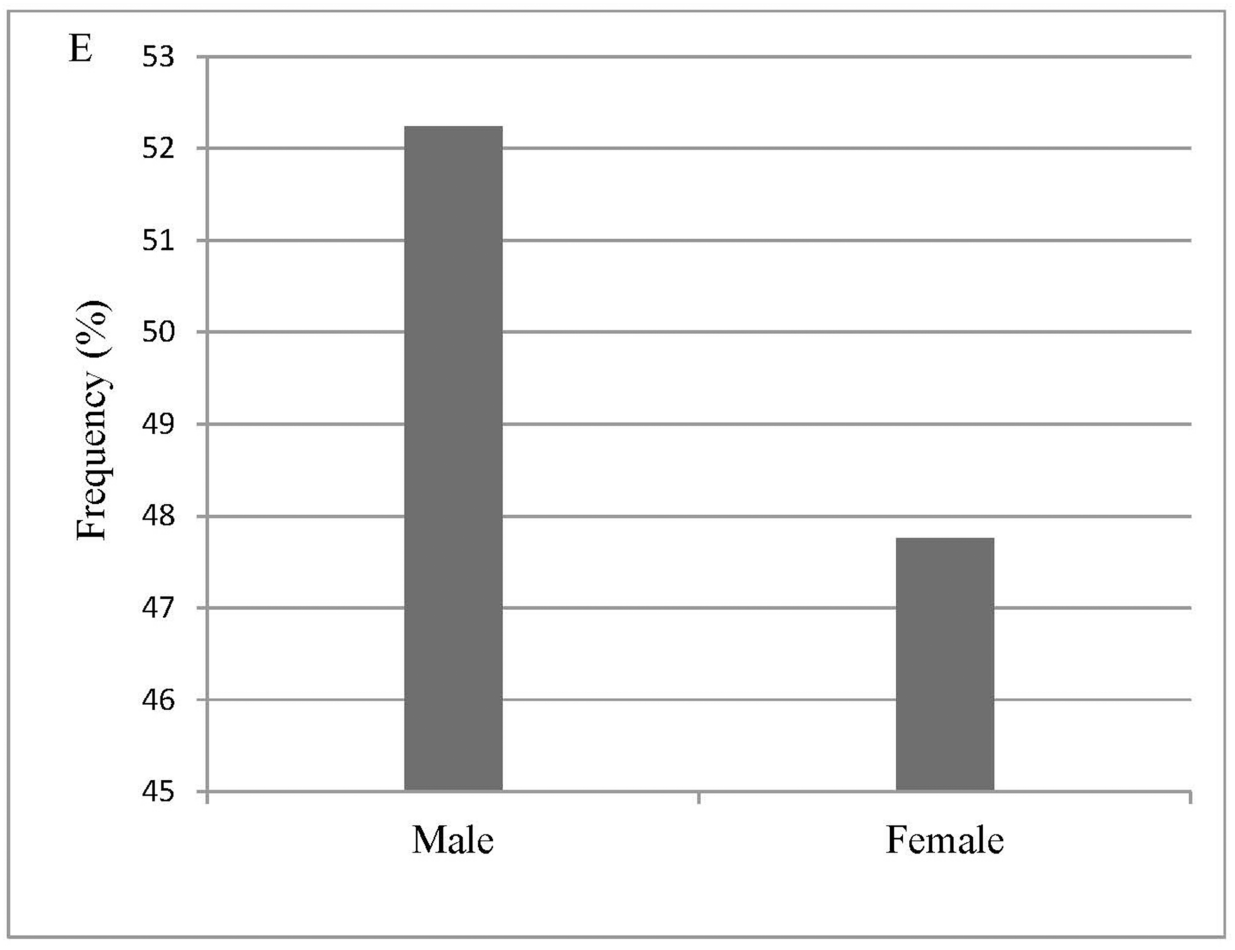
Gender.

Based on these criteria, we select 30 videotapes that record more than one hundred individuals (https://data.qdr.syr.edu/dataset.xhtml?persistentId=doi:10.5064/F651PANT). We summarize the general characteristics and conditions experienced by the selected more than one hundred individuals, specifically seismic intensity according to the CSIS (Fig 1), social context (Fig 2), the year in which the earthquakes occurred (Fig 3), the specific earthquake (Fig 4), and gender (Fig 5).

### Analysis methods

#### Qualitative analysis

The videotapes can objectively and accurately record the entire emergency response process. The entire process of a single individual is reflected by his or her individual characteristics, pre-earthquake status and response behaviors [33]. Therefore, we can obtain raw material and data by scientifically analyzing a large number of videos of earthquake evacuations.

The Transana software package (http://www.transana.org/) provides a platform that can be used to analyze the EERB of each individual [2,34]. It can also be used to extract keyword groups and keywords that could accurately code the entire emergency response process of a pedestrian (Table 1). The main interface of Transana comprises four parts: the Sound Window, Video Window, Transcript Window, and Data Window [35].

**Table 1:** Terms used in Transana.

	Transana term	Description
Raw media file	Series	The 30 selected videotapes recorded in mainland China
	Episode	A videotape of the 30 selected videotapes (e.g., 20080512 Wenchuan earthquake-1)
	Transcript	A videotape may contain more than one individual, but the transcript represents the EERB of a single individual shown in this videotape (e.g., 20080512 Wenchuan earthquake-1-1(raw video data))
Coded media file	Collection	One individual’s EERB is encoded as a collection, and a collection includes one or more clips (e.g., 20080512 Wenchuan earthquake-1-1 (coded EERB data))
	Clip	Each clip represents an action that constitutes part of the emergency response, such as taking cover or evacuating
Coding structure	Keyword group	Includes one or more keywords

Several transcripts are created for each videotape that features more than one individual. Each transcript represents the earthquake emergency response mode of an individual and his or her sequence of actions [20]. When a time code is inserted, the transcripts and videotape can be matched to achieve synchronous playback.

The software creates a clip when one individual changes his or her response behavior. A collection includes all clips that correspond to that individual. By encoding the transcripts of more than one hundred individuals, we extracted the keyword groups and the keywords of individual characteristics, the pre-earthquake phase and each pedestrian’s response phase.

#### Quantitative analysis

The Tracker software package (http://physlets.org/tracker/) provides a quantitative method for analyzing entire emergency responses. For each evaluated experimental data, we provide the minimum and maximum values, average value, median, arithmetic mean and standard deviation. In this paper, the used method was previously provided by other studies [2].

Before analyzing a videotape, we must calibrate it. The primary steps involved in using the Tracker software package are as follows.

1. Set the Start frame, End frame and Step size that we aim to analyze.
2. Calibrate the distorted shape and the scale.
3. Set the reference frame origin and angle.
4. Track individuals. Each individual in the videotape is marked as a point mass with different colors and shapes and the point mass center is located at the individuals’ shoulder level. Then, his or her position at each Step size can be marked. Finally, the evacuation route of each individual is represented by a line that has the same color as the point mass.
5. Plot and analyze the tracks. Each individual’s instantaneous values of speed can be obtained at each Step size. The Tracker software package can display graphs of each individual’s speed.
6. The definition of the average evacuation speed (${\text{v}}_{j}^{T_{j}}$) of an individual (*j*) throughout his or her entire emergency evacuation process is as follows:
  *N* individuals are shown in a single videotape. The corresponding set is {ind(1), ind(2)⋯ind(*j*)⋯ind(*N*)}. The duration of the entire emergency evacuation process of an individual (*j*) is *T*_*j*_, the step size is *L*,$\text{k}\left(j\right)=\frac{T_{j}}{L}$, and the set of time points used in calculating the instantaneous speed of an individual (*j*) is {t(1), t(2)⋯t(*i*)⋯*t*(*k*(*j*))}, i∈{1,2,⋯*k*(*j*)}. At time point *t*(*i*), the instantaneous speed of an individual is ${\text{v}}_{j}^{t\left(i\right)}$, and the corresponding set is $\left\{{\text{v}}_{j}^{t\left(1\right)},{\text{v}}_{j}^{t\left(2\right)}\cdots {\text{v}}_{j}^{t\left(i\right)}\cdots {\text{v}}_{j}^{t\left(k\left(j\right)\right)}\right\}$.
  The average speed of an individual (*j*) during the entire emergency evacuation process is given by the following:
  $${\text{v}}_{j}^{T_{j}}=\frac{1}{k\left(j\right)}\sum_{i=1}^{k\left(j\right)}v_{j}^{t\left(i\right)}\;\text{i}\in \left\{1,2,\cdots k\left(j\right)\right\},j\in \left\{1,2,\cdots N\right\}.$$ (1)
  According to Eq (1), $v_{j}^{t\left(i\right)}$ could be evaluated using the displacement of an individual over a short time interval Δ*t*' (*i*) around *t*(*i*):
  $$v_{j}^{t\left(i\right)}=\frac{\left\|\vec{\text{x}}\left(t\left(i\right)+\Delta t^{\prime }\left(i\right)/2\right)-\vec{\text{x}}\left(t\left(i\right)-\Delta t^{\prime }\left(i\right)/2\right)\right\|}{\Delta t^{\prime }\left(i\right)}.$$ (2)
  Eq (2) was previously provided by other studies [2,36].
7. At time point t_z_ (*i*), the average instantaneous evacuation speed (${\text{v}}_{}^{t_{\text{z}}\left(i\right)}$) of all individuals who are evacuating in a single videotape is defined as follows.
  At time point t_z_ (*i*), the total number of individuals who are evacuating is *M* (*i*), and the corresponding set is {*ind*_*eva*_ (1), *ind*_*eva*_ (2)⋯*ind*_*eva*_ (*j*)⋯*ind*_*eva*_ (*M* (*i*))}, where *eva* denotes the evacuation status. The time required for all individuals to evacuate out of the room in the videotape is *T*_z_, the step size is *L*, and the set of time points used in calculating the instantaneous speeds of individuals is {t_z_ (1), t_*z*_ (2)⋯t_*z*_ (*i*)⋯*t*_z_ (*k*)},$\text{k}=\frac{T_{\text{z}}}{L}$. At t_z_ (*i*), the instantaneous evacuation speed of each individual is ${\text{v}}_{ind_{eva}\left(j\right)}^{t_{\text{z}}\left(i\right)}$, j ∈ {1,2,⋯*M*(*i*)}, i ∈ {1,2,⋯*k*}.
  At t_z_ (*i*), the function is
  $${\text{v}}_{}^{t_{\text{z}}\left(i\right)}=\frac{1}{M\left(i\right)}\sum_{j=1}^{M\left(i\right)}v_{ind_{eva}\left(j\right)}^{t_{z}\left(i\right)},j\in \left\{1,2,\cdots M\left(i\right)\right\},i\in \left\{1,2,\cdots k\right\}.$$ (3)

## Results and discussion

This paper makes the following contributions and discusses the following differences between this new study and previous studies.

1. Little research has been conducted on the emergency responses of crowds during earthquake disasters. Models used in the study of earthquake emergency responses represent a complex nonlinear physical problem [37]. Due to their severe destructive effects, earthquake disasters damage buildings and affect evacuation routes. Therefore, the theory and models that describe emergency evacuation during fires and other disasters cannot be applied to earthquake disasters. It is of great significance to study the EERB of pedestrians.
2. In this paper, we used Transana software to build a Keyword database of pedestrian evacuation (i.e., Individual characteristics, Pre-earthquake phase and Pedestrian response phase). This keyword database is used to analyze the decision-making process of pedestrian evacuation and the pattern of evacuation behavior.
3. In the analysis of Delay time, the influence of seismic intensity on the delay time of pedestrians is analyzed. Then, we construct a seismic intensity-gender matrix of the average delay time. From this matrix, we can determine the average delay time for men and women under different seismic intensities.
4. In the analysis of Evacuation speed, we discuss the correlation between evacuation speed and seismic intensity in a location (density: person/m^2^).
5. One innovation of this paper is the analysis of the influence of seismic intensity on pedestrian evacuation.
6. In the analysis of Evacuation route choices and Emergency exit choices, the four stages of the pedestrian evacuation process, the factors affecting the evacuation route and the emergency exit selection are analyzed. In this paper, we also analyze the effects of room layout on the efficiency of evacuation.

The required database of the EERB of pedestrians is composed of the following seven parts.

### Individual characteristics

Based on previous studies and the limitations of videotapes, this paper divides individual characteristics into one keyword group: gender (Table 2).

**Table 2:** Individual characteristics, according to gender.

	Keyword group	Keyword	Frequency (%)	Description
Individual characteristics	Gender	Male Female	52.24 47.76	Some differences in physiological and psychological characteristics exist between males and females. These differences are reflected in the EERB of pedestrians [38–39].

### Pre-earthquake phase

The pre-earthquake status of individuals includes location, social context and action (Table 3). These factors affect the emergency response decisions of pedestrians. The three keyword groups are open groups that include not only existing keywords but also new keywords that may arise as the number of new earthquakes increases.

**Table 3:** Pre-earthquake status according to location, social context and action.

	Keyword group	Keyword	Frequency(%)	Description
Pre-earthquake phase	Location (an open keyword group)	In a classroom	25.37	After an earthquake, people display different EERB in different locations [5,40–41].
		In an office	14.93	
		In an Internet cafe	13.43	
		In a library	8.21	
		In a periodical reading room	7.46	
		In a hallway	5.97	
		In a supermarket	5.22	
		In a computer room	3.73	
		At home	2.98	
		At a barbershop	2.98	
		In an e-reading room	2.24	
		At a hospital	2.24	
		In a bank	1.50	
		At an airport	1.50	
		In a mobile phone shop	1.49	
		In an auto repair factory	0.75	
		NK(The new keyword)	NK	
	Social context (an open keyword group)	With colleagues	26.12	According to previous studies [42–43], people are easily influenced by others during their responses to earthquake emergencies.
		With classmates	25.37	
		Alone	20.15	
		With strangers	11.94	
		With family	7.46	
		With friends	6.72	
		With patients	2.24	
		NK	NK	
	Action (What were they doing when the earthquake occurred?) (an open keyword group)	Working	27.61	The pre-earthquake actions taken by pedestrians may affect their perceptions. For example, pedestrians who are moving cannot easily feel slight shaking. In contrast, pedestrians who are still can easily feel slight shaking and take protective action [44–45].
		Studying	25.37	
		Surfing the Internet	14.18	
		Keeping shop	9.70	
		Reading	7.46	
		Reading periodicals	6.72	
		Walking	3.73	
		Doing housework	2.99	
		Chatting	1.49	
		Sleeping	0.75	
		NK	NK	

### Pedestrian response phase

Pedestrians’ behaviors in indoor environments have a significant influence on inhabitants’ safety at different evacuation stages. Hence, an important issue is understanding how people interact with other individuals and with the environment modified by the earthquake. The EERB of pedestrians include first response behaviors, first protective behaviors, and subsequent protective behaviors (Table 4). These keyword groups are also open groups.

**Table 4:** Pedestrian response behaviors.

	Keyword group	Keyword	Frequency (%)	Description
Pedestrian response phase	First response behaviors (an open keyword group)	Look around	31.33	When pedestrians feel the ground shake, due to the uncertain nature of individual perceptions, pedestrians are often not sure what happened, and they need to obtain information from the environment or other people to confirm whether an earthquake has occurred. They look around and communicate with others to determine what protection behavior is most appropriate [19,41,45].
		Take self-protective action	24.63	
		Continue performing the original task	14.18	
		Continue performing the original task + Look around	11.19	
		Look around + Oral communication	10.45	
		Oral communication	4.48	
		Inaction	2.99	
		Provide assistance to others	0.75	
		NK	NK	
	First protective behaviors (an open keyword group)	Evacuate	49.24	After confirming that an earthquake is occurring and exhibiting response behaviors, people display their first protective behavior. Evacuate: Based on their survival instincts, people are eager to move to safe places. After a series of decisions (e.g., selecting evacuation routes and exits), people escape from the buildings that they occupy. Hold one’s head+ squat down+ hide under a nearby desk: because of previous emergency drills, students can be guided by teachers to first hide under nearby desks. Rush into a building, use a telephone, wait for family: as a result of kinship, people tend to find their families and evacuate with them. Hold on to a door frame, hold on to a wall: it is difficult for people to maintain their balance when the ground is shaking strongly. They must hold on to a door frame or hold on to a wall. Continue performing the original task, pack up one’s belongings: people need to work and get their belongings [37, 38,44].
		Hold one’s head + Squat down + Hide under a nearby desk	23.88	
		Try to evacuate	8.21	
		Continue performing the original task	5.97	
		Hold on to a door frame + Hide under the door frame	5.22	
		Provide assistance to others	2.24	
		Pack up one’s belongings	1.49	
		Hold on to a wall + Hide	0.75	
		Wait for family members	0.75	
		Telephone others + Evacuate	0.75	
		Look for safe positions indoors	0.75	
		Rush into a building	0.75	
		NK	NK	
	Subsequent protective behaviors (an open keyword group)	Evacuate	32.84	Stop hiding + evacuate: when the ground shakes strongly, people tend to find a safe area in the room and hide. When the degree of ground shaking has diminished, they evacuate from the building [44,46]. Wait for family + evacuate with them: when pedestrians know that their family and friends are nearby, they tend to find them and evacuate from buildings with them. Return room to collect belongings: after escaping from the building, some people return to the building to get their belongings and then leave the building again.
		Stop hiding + Evacuate	8.21	
		Return to the room to collect belongings	5.97	
		Wait for family members + Evacuate with them	3.73	
		Look around + Evacuate	2.24	
		Return to do one’s original task	0.75	
		NK	NK	

**Notes**: In this table, "+" indicates that the two actions occur simultaneously, and "NK" indicates the new keywords that may arise by increasing the number of new earthquakes. The behaviors we discuss in this paper are indoor scenarios behaviors.

Improving the efficiency of emergency response is closely related to the decisions of pedestrians. During earthquake emergency responses, the most effective self-protection behavior is to evacuate from buildings or find a safe position before the buildings collapse. Evacuation is influenced by many environmental factors and the presence of other individuals. Finding safe positions is based on the geometry of the environment, the level of damage to the environment and social factors [32]. Of course, in an actual earthquake emergency response, many other behaviors are involved.

**Fig 6:**
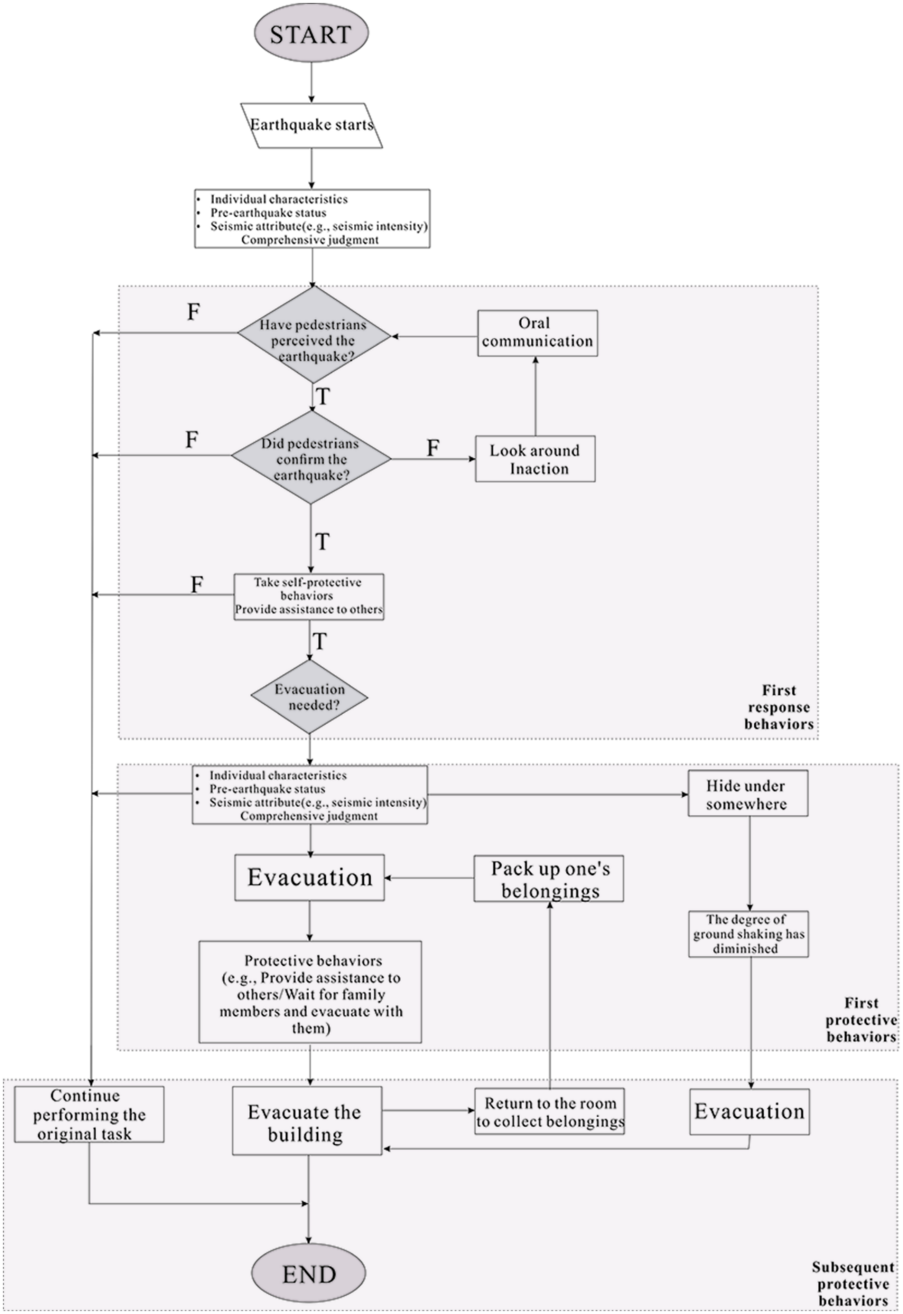
General decision flowchart of pedestrians in earthquake.

Fig 6 shows the general decision flowchart of pedestrians using the chronological organization of the EERB expressed in Tables 2–4. The process of first response behaviors, first protective behaviors and subsequent protective behaviors are evidenced [32].

### Delay time

**Fig 7:**
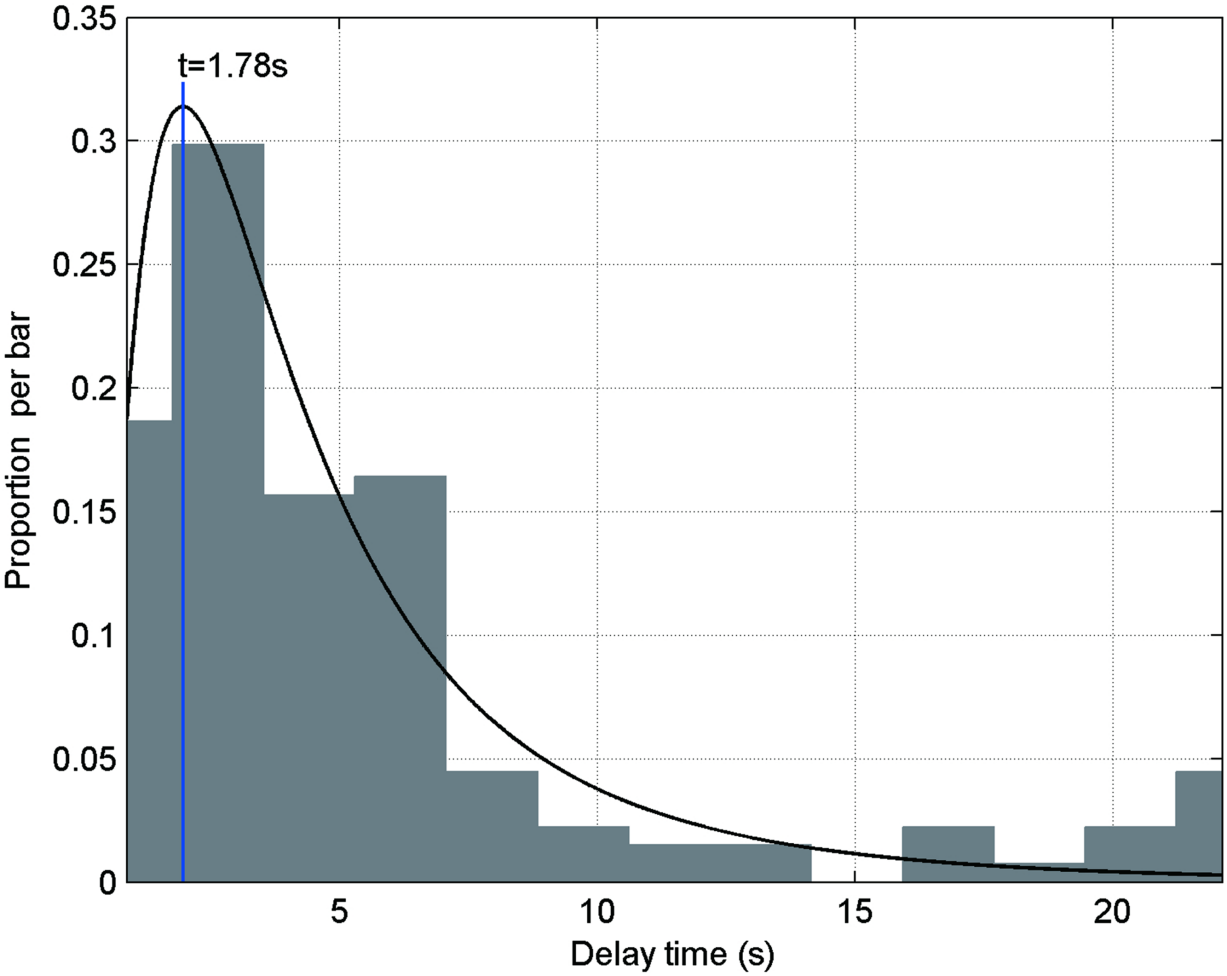
Delay time distribution and the related log-normal curve: The arithmetic mean is 5.44 s; the std. deviation is 0.82; and the highest frequency of delay time is 1.78 s.

Delay time refers to the time elapsed from the occurrence of the earthquake to the first protection behavior and includes both the recognition time and the response time. The data distribution of delay time follows a log-normal curve. Fig 7 shows delay time distributions and related curves: a log-normal curve could be suggested for the indoor scenarios.

Data for delay time are various and affected by many factors. Eqs (4) and (5) propose the relationship among the parameters that are considered by a pedestrian when making an evacuation decision:

$${\vec{T}}^{delay}=f\left(Si,Lo,Ac,Sc,Ed\text{,}Age,Gender\right)$$ (4)

$$\text{min}{\vec{T}}^{delay}$$ (5)

Seismic intensity is one of the factors that exerts a paramount influence on the delay time. In this paper, we discuss only the correlation between the delay time and the seismic intensity in terms of gender. The data of delay time are divided into six categories, based on seismic intensity used in the CSIS (I-IV, V, VI, VII, VIII, and IX).

**Fig 8:**
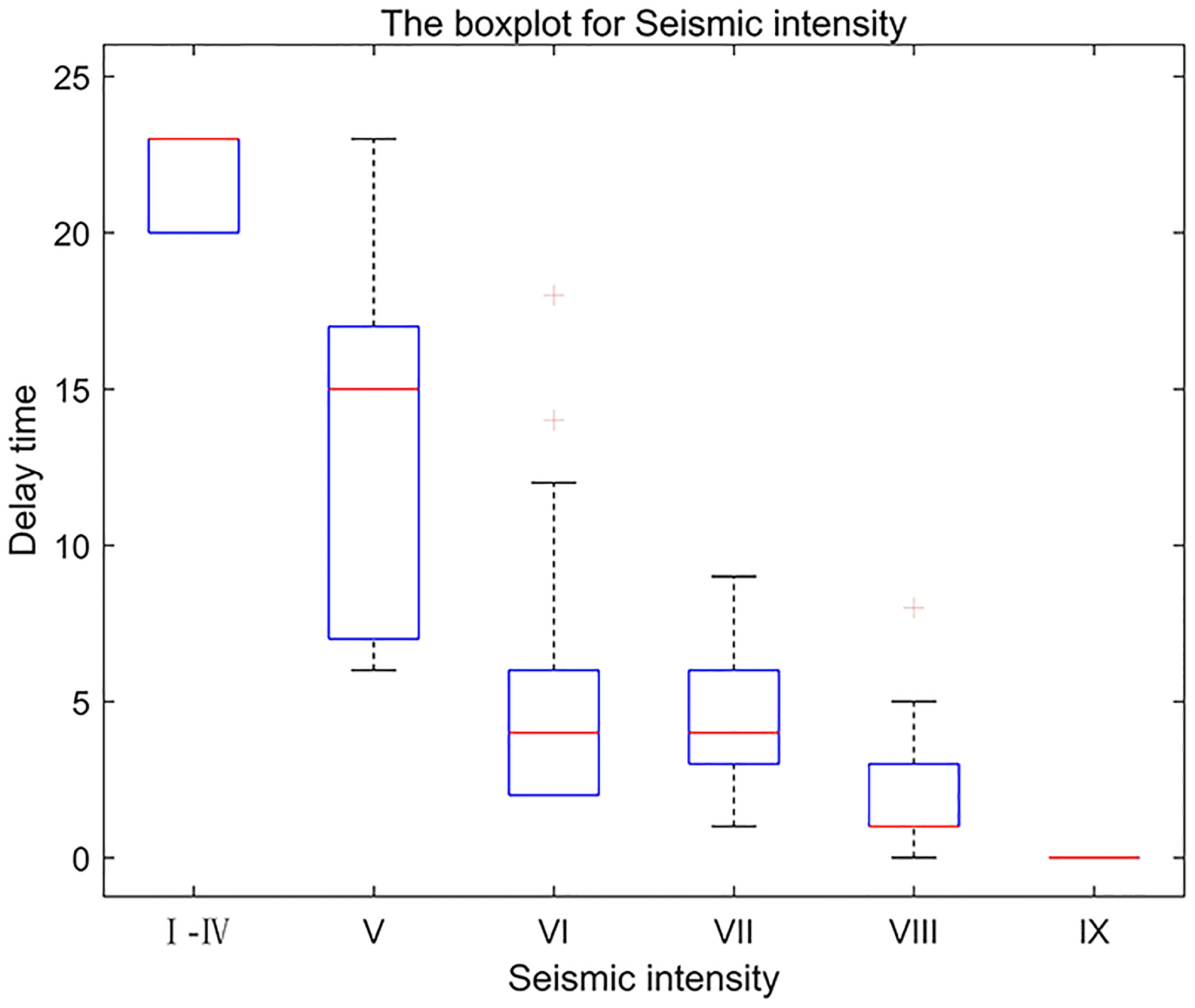
The boxplot for each seismic intensity class used in the CSIS (I-IV, V, VI, VII, VIII, and IX).

Based on the data in Table 5, we have plotted a boxplot for each seismic intensity class (Fig 8). The average delay time for intensitiesI-IV is 21.71 s. This result occurs because the ground shaking is weak in this seismic intensity range. Thus, pedestrians do not clearly perceive the earthquake. The pedestrians must obtain more information from the environment and the people around them to determine whether an earthquake has occurred and what type of protective action must be taken. Table 5 shows that the average delay time under intensities of I-IV and V are relatively long (21.71 s and 13.80 s, respectively). However, the average delay time under VI decreases sharply to 4.97 s. In this interval, the average delay time undergoes a mutation. Under the intensities of VI-IX, the average delay time of pedestrian decreases gradually from 4.97 s to 0.00 s. Based on the above analysis, we conclude that the delay time increasingly shortens as the ground shaking intensifies.

**Table 5:** Delay time, according to seismic intensity.

Seismic intensity	Numerical range (s)	Average value (s)	Median (s)	Std. deviation	Sample dimension
I-IV	20.00–23.00	21.71	23.00	1.60	7
V	6.00–23.00	13.80	15.00	6.37	10
VI	2.00–18.00	4.97	4.00	3.77	35
VII	1.00–9.00	4.57	4.00	2.20	44
VIII	0.00–8.00	1.78	1.00	1.53	36
IX	0.00–0.00	0.00	0.00	0.00	2

**Fig 9:**
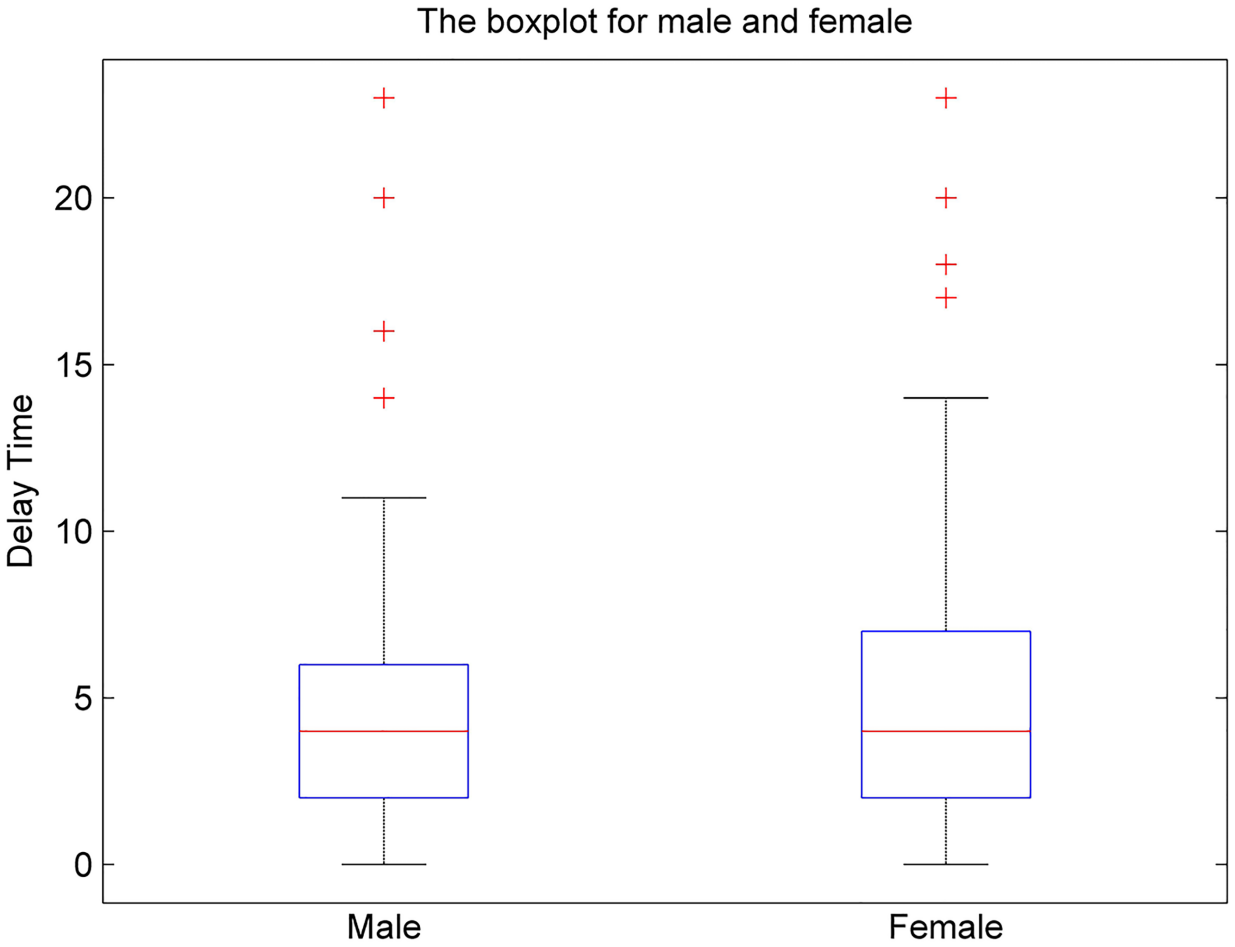
The boxplot of delay time between male and female.

Furthermore, this paper has also studied the relationship between delay time and gender. We have created a Figure with two boxplots of the delay time: one for male and one for female. As shown in Fig 9, gender does not influence delay time. The average delay time of females is only 0.40 s longer than that of males. And, the effect of gender on delay time is not distinct.

**Fig 10:**
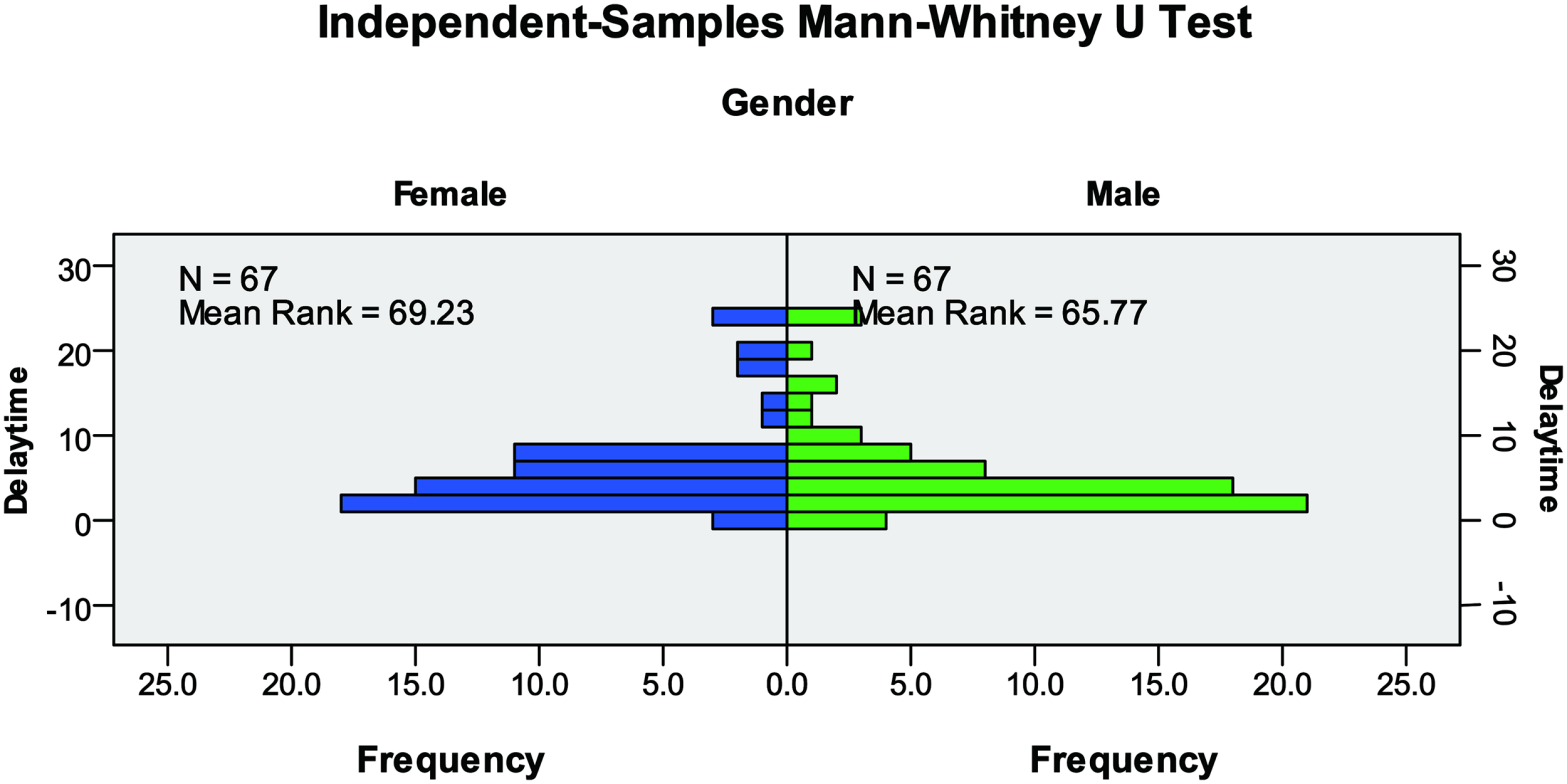
Independent-samples Mann-Whitney U Test.

This article has used the Mann-Whitney U-test to investigate the impact of gender on delay time (Fig 10). Based on the results of the Mann-Whitney U-test (P = 0.604>0.05: Retain the original hypothesis), we can see that the distribution of delay time is the same across categories of gender. Thus, there is no difference between the delay time values for male and female evacuees.

Based on the analysis presented above, we construct a seismic intensity-gender matrix of the average delay time (Table 6). From this matrix, we can determine the average delay time for the two genders under different seismic intensities.

**Table 6:** Seismic intensity-gender matrix of the average delay time.

Seismic intensity	Males		Females	
	Average value (s)	Numerical range (s)	Average value (s)	Numerical range (s)
IIV	22.00	20.00–23.00	21.50	20.00–23.00
V	16.00	9.00–23.00	12.33	6.00–23.00
VI	4.83	2.00–14.00	5.27	2.00–18.00
VII	4.35	1.00–9.00	4.70	1.00–8.00
VIII	1.82	1.00–5.00	1.74	0.00–8.00
IX	0.00	0.00–0.00	0.00	0.00–0.00

### Evacuation speed

Evacuation speed is a crucial parameter used in evacuation models. It is directly related to whether crowds can safely evacuate to designated areas within a specified time. Currently, few quantitative studies investigate the speed of earthquake evacuation. This lack of research is because earthquakes are not repeatable, and it is difficult to reconstruct pre-earthquake conditions after an earthquake. Moreover, post-earthquake scenes are complex, and available analytical data are limited.

In this paper, the evacuation scenarios we discuss are indoor scenarios with a large number of individuals. The data distribution of instantaneous evacuation speed follows a log-normal curve, which is consistent with the findings of previous studies in indoor scenarios with a high number of individuals [2].

**Fig 11:**
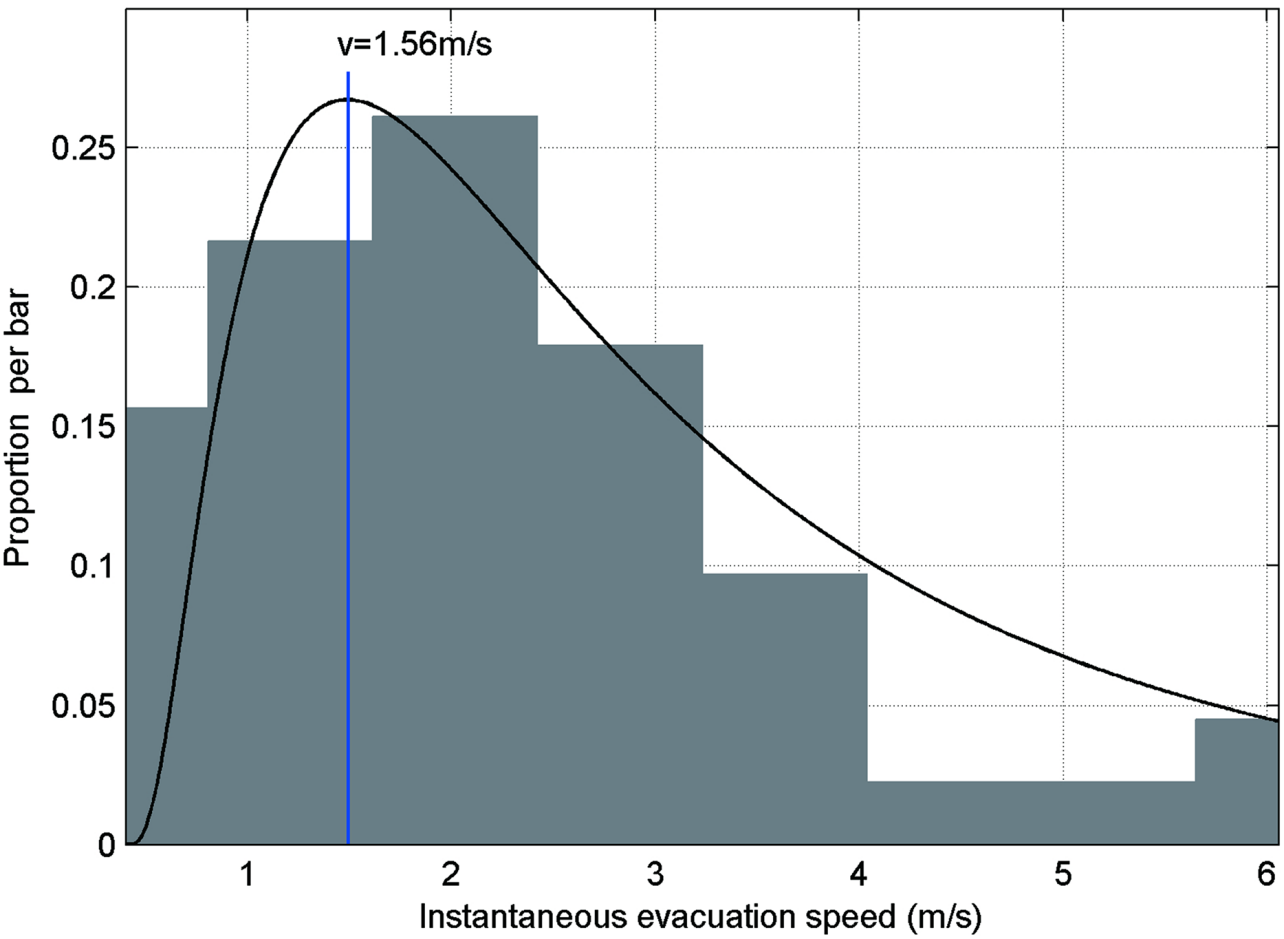
Instantaneous evacuation speed distribution and the related log-normal curve. The arithmetic mean is 2.21 m/s; the std. deviation is 0.66; and the highest frequency of speed is 1.56 m/s.

Fig 11 shows instantaneous evacuation speed distributions and the related log-normal curve.

According to previous studies, evacuation speed is affected by many factors, such as seismic attributes, building characteristics and individual characteristics. Each of these three factors can also be divided into several aspects. In this paper, we discuss only the correlation between evacuation speed and seismic intensity in a location (density: person/m^2^).

In terms of the effects of location (density: person/m^2^) on evacuation speeds, the more crowded a location, the slower the evacuation speed [2,47]. Generally, classrooms are densely populated places. There are typically 50 students and two emergency exits in classrooms in China. However, the back exit of the classroom is not always open. After an earthquake, many students congregate near the front exit of the classroom, producing an arch-like formation that blocks the exit. The evacuation speed of students decreases sharply, and the minimum instantaneous evacuation speed is approximately 0.66 m/s. In places where personnel are scarce, and the space is wide (such as hallways), the evacuation speeds of pedestrians are faster, and the maximum instantaneous evacuation speed is approximately 6.96 m/s.

In each row of Table 7, the average evacuation speed increases from I-IV to IX. In each column, the average evacuation speeds observed in various places were sorted in ascending order. The average evacuation speed reported for barber shop is 0 because the staff of the barber shop investigated in this study did not evacuate; instead, they continued engaging in their original work. Based on the above research, we conclude that the average evacuation speed increases with increasing seismic intensity and decreases with increasing population density. In this matrix, the value displayed in the lower right corner is the largest, and the value displayed in the upper left corner is the smallest.

**Table 7:** The seismic intensity—Location (density: person/m^2^) matrix of the average evacuation speed.

Location (person/m2) (average population density in each location)	Seismic intensity					
	I-IV	V	VI	VII	VIII	IX
Barber shop	0	/	/	/	/	/
Classroom (1.03)	/	1.11	/	0.91	1.23	/
Hospital (0.85)	/	1.40	/	/	/	/
Internet cafe (0.77)	/	/	/	1.49	/	/
E-reading room (0.69)	/	/	1.63	/	/	/
Computer room (0.62)	/	/	/	1.65	/	/
Periodical reading room (0.60)	/	/	1.69	/	/	/
Bank (0.59)	/	/	/	/	/	1.73
Library (0.58)	/	/	1.86	/	/	/
Supermarket (0.55)	0	1.12	1.95	2.47	/	/
Office (0.41)	/	/	1.47	1.55	2.12	/
Airport (0.32)	/	/	/	2.05	/	/
Home (0.31)	/	2.55	/	/	/	/
Hallway (0.28)	/	/	1.97	/	3.31	/
Mobile phone shop (0.23)	/	/	/	2.55	/	/
Auto repair factory (0.21)	/	3.27	/	/	/	/

**Fig 12:**
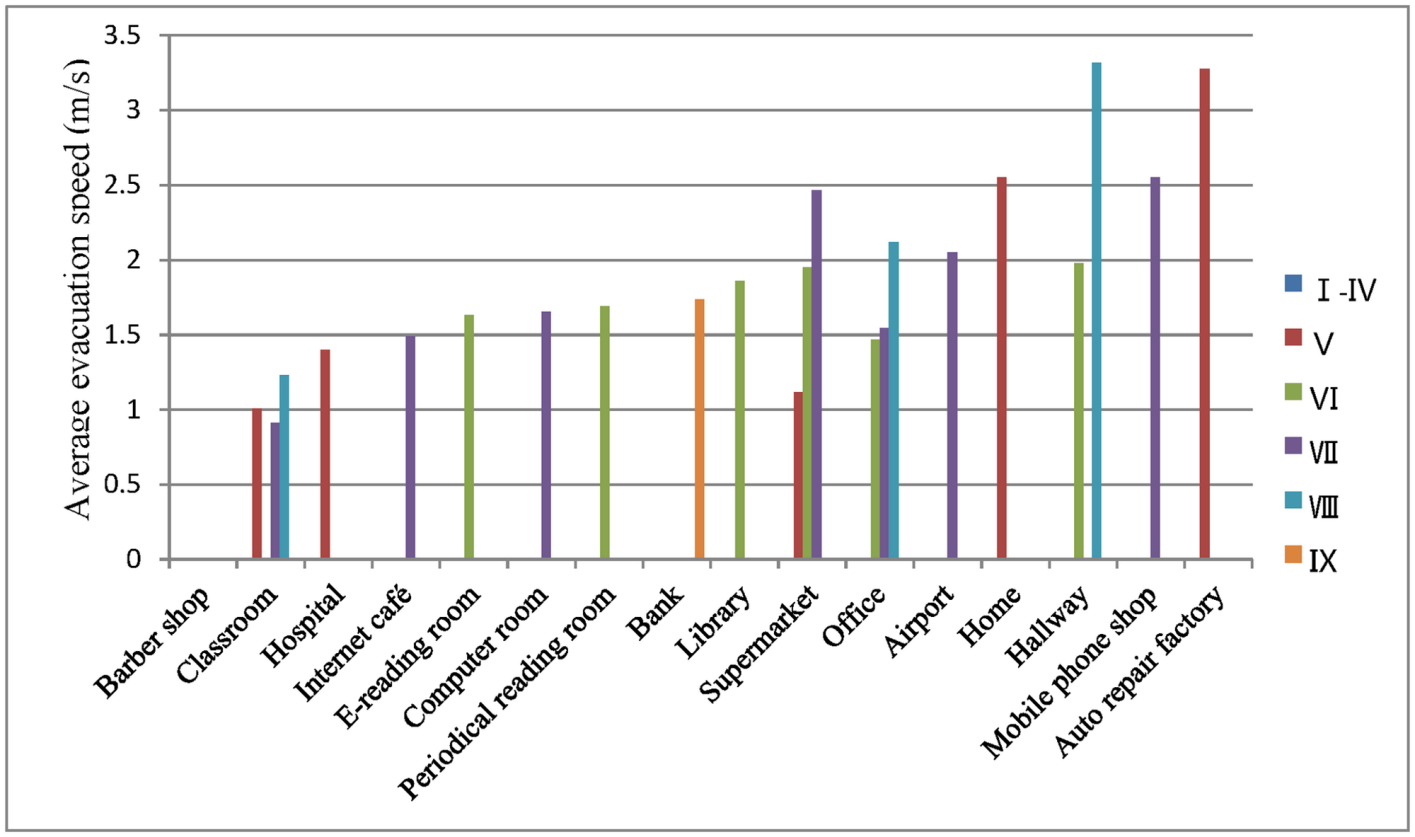
Average evacuation speeds for different seismic intensities and locations.

**Fig 13:**
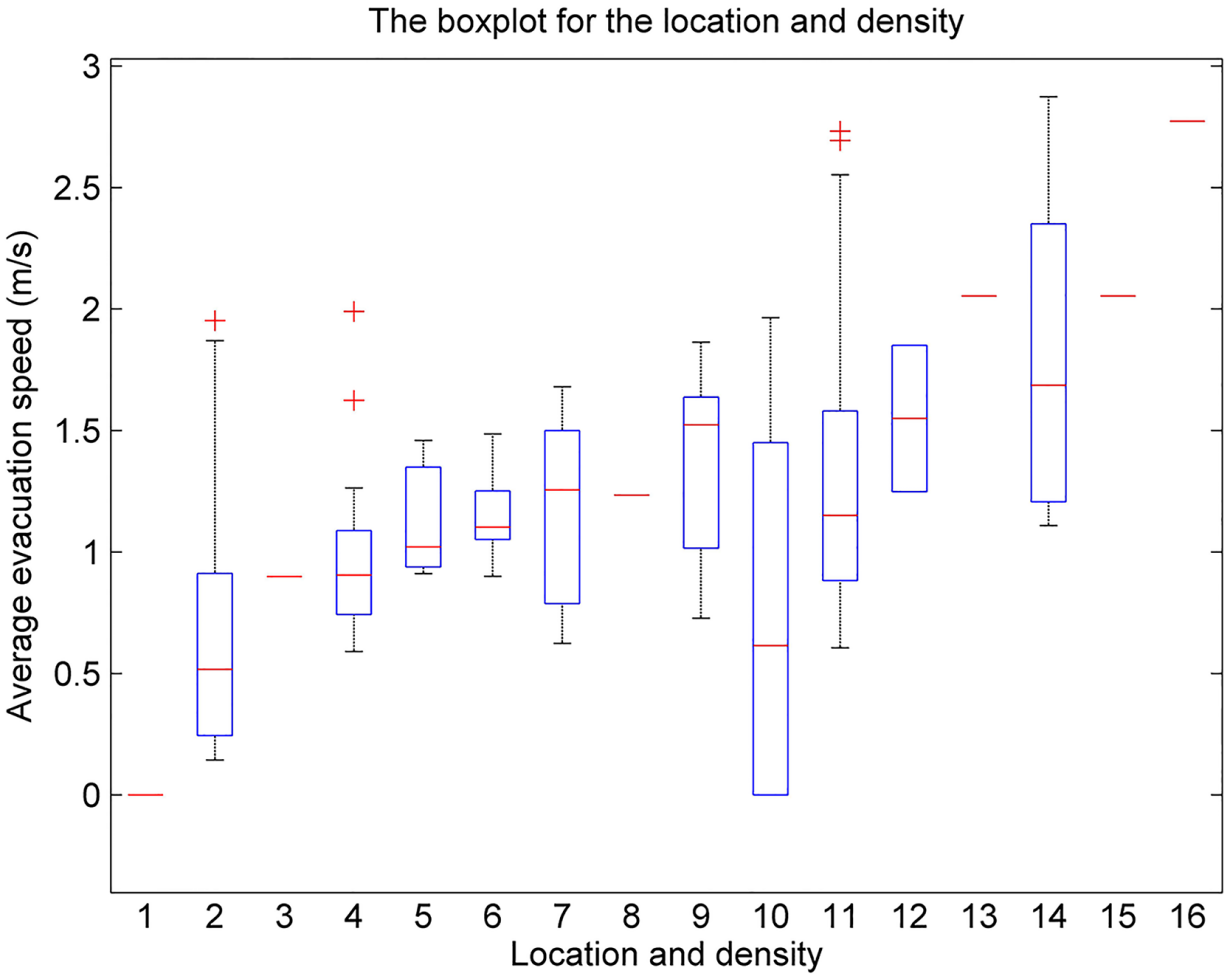
A box plot of the different locations (density: person/m2). **Notes: 1**—Barber shop, 2-Classroom (1.03), 3-Hospital (0.85), 4-Internet cafe (0.77), 5-E-reading room (0.69), 6-Computer room (0.62), 7-Periodical reading room (0.60), 8-Bank (0.59), 9-Library (0.58), 10-Supermarket (0.55), 11- Office (0.41), 12- Airport (0.32), 13- Home (0.31), 14- Hallway (0.28), 15- Mobile phone shop (0.23), and 16- Auto repair factory (0.21).

This matrix is represented as a histogram (Fig 12). We have plotted box plots for the data in Table 7 (Fig 13). The numerical range, average value, median, and variance of the delay time and evacuation speed for different genders, ages, and locations are shown in Table 8.

**Table 8:** Numerical range, average value, median, and the std. deviation of the delay time and evacuation speed for different gender and location.

Influencing factor	Delay time (s)				Evacuation speed (m/s)			
Gender	Numerical range	Average value	Median	Std. deviation	Numerical range	Average value	Median	Std. deviation
Male	0.00–23.00	5.24	3.00	5.54	0.74–3.37	1.64	1.60	0.35
Female	0.00–23.00	5.64	3.00	5.85	0.64–3.25	1.62	1.41	0.47
**Location**								
At a barbershop	23.00–23.00	23.00	23.00	0.00	0.00–0.00	0.00	0.00	0.00
In a classroom	1.00–9.00	1.88	1.00	1.57	0.64–2.45	1.12	1.02	0.70
At a hospital	6.00–7.00	6.67	7.00	0.58	1.40–1.40	1.40	1.40	0.00
In an Internet cafe	3.00–9.00	5.22	4.50	2.10	1.09–2.49	1.49	1.41	0.12
In an e-reading room	2.00–2.00	2.00	2.00	0.00	1.41–1.96	1.63	1.52	0.08
In a computer room	4.00–8.00	5.60	5.00	1.82	1.40–1.99	1.65	1.60	0.05
In a periodical reading room	2.00–14.00	5.50	5.00	3.54	1.12–2.18	1.69	1.76	0.13
In a bank	0.00–0.00	0.00	0.00	0.00	1.73–1.73	1.73	1.73	0.00
In a library	2.00–18.00	5.46	5.00	4.52	1.23–2.36	1.86	2.02	0.15
In a supermarket	2.00–23.00	13.57	20.00	9.09	1.12–2.47	1.87	1.95	0.31
In an office	0.00–12.00	3.70	3.00	3.25	1.11–3.23	1.88	1.65	0.52
At an airport	8.00–8.00	8.00	8.00	0.00	1.75–2.35	2.05	2.05	0.18
At home	14.00–17.00	15.75	16.00	1.26	2.55–2.55	2.55	2.55	0.00
In a hallway	2.00–8.00	3.38	2.00	2.20	1.61–3.37	1.31	2.19	0.48
In a mobile phone shop	6.00–6.00	6.00	6.00	0.00	2.55–2.55	2.55	2.55	0.00
In an auto repair factory	23.00–23.00	23.00	23.00	0.00	3.27–3.27	3.27	3.27	0.00

### Evacuation route choices

In earlier studies of the effects of room layout on evacuee choices, Lovreglio et al. [48]used a mixed logit model and found that the presence of smoke, emergency lighting, distance of exit, number of evacuees near the exits and the decision-maker and flow of evacuees through the exits significantly affect local exit choice; then, Lovreglio et al.[49]used a binary logit model to analyze the occurrence of herding behavior and found that the occurrence of herding behavior is affected by both environmental and personal factors. In particular, the model shows that the personal tendency to engage in herding behavior can play a key role in selecting an exit. Yang et al.[50] analyzed surveillance videos that recorded the evacuation of students during the Wenchuan earthquake and found that the relation between the arrival time and the order in which students arrive is nonlinear. In our paper, we have compared the effects of factors such as the role of teachers and the width of the evacuation path on the efficiency of evacuation.

In this paper, we analyze the evacuation route chosen by students in a classroom in the No. 2 Middle School of Ganqika (20130422 Tongliao earthquake-Ganqika). Each student in the classroom is marked as a point mass with a different color and shape, and the point mass center is located at the students’ shoulder level. The position of each student is noted in every four frames from the moment they leave their seat. Thus, all students are traced until they reach the emergency exit.

**Fig 14:**
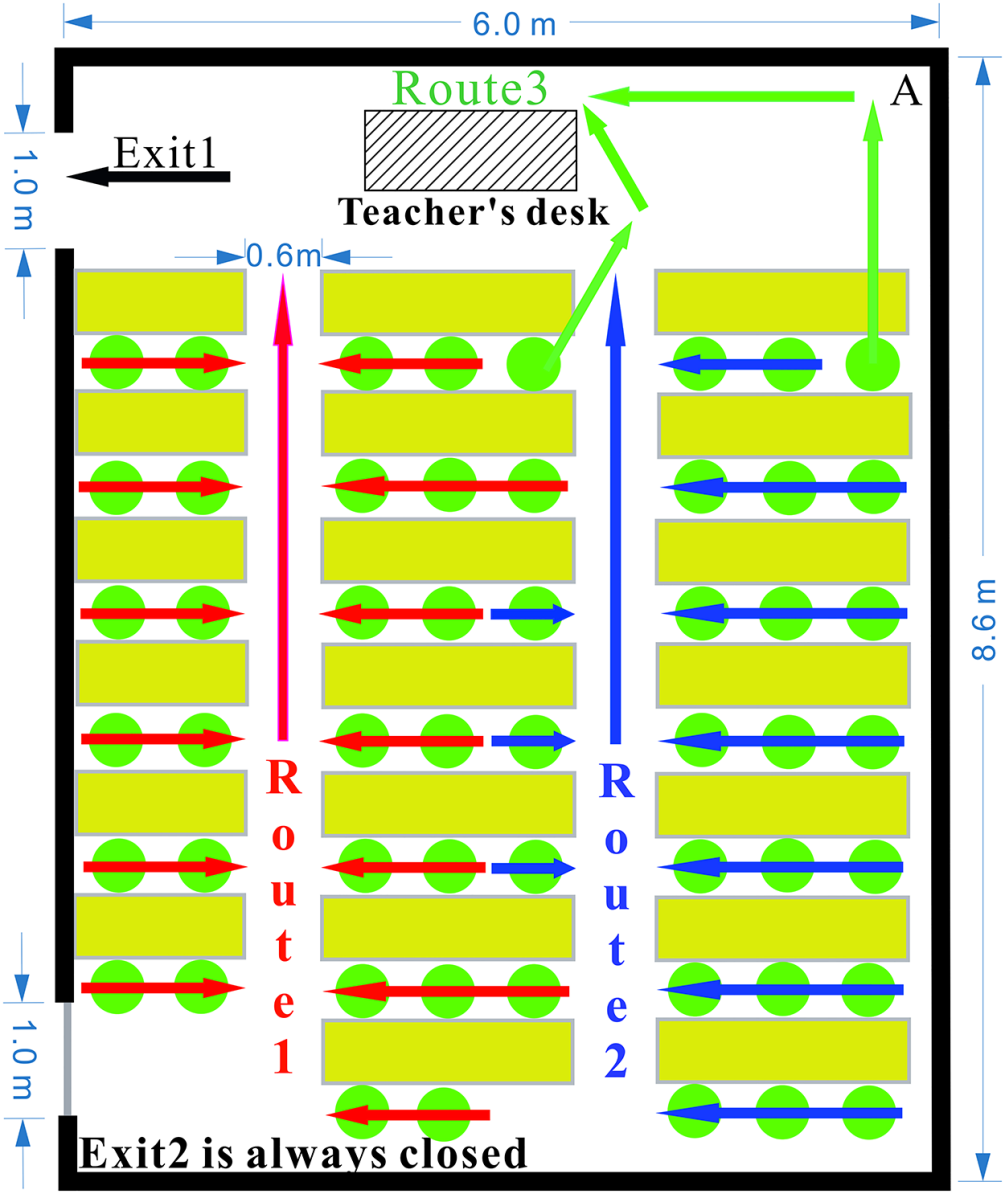
The classroom layout and evacuation trajectories in the No. 2 Middle School of Ganqika.

The classroom layout and evacuation trajectories are shown in Fig 14. There are 53 students in the classroom, which has two emergency exits and three evacuation routes. However, Exit 2 is always closed. According to Fig 14, students tend to choose evacuation routes close to the emergency exit. Two students close to Route 2 choose to evacuate from the classroom via Route 1, which is near the emergency exit. Twenty-eight students (red arrows) evacuate from the classroom using Route 1, whereas twenty-three students (blue arrows) use Route 2. Two students (green arrows) evacuate from the classroom using Route 3, which is a shortcut to the emergency exit.

**Fig 15:**
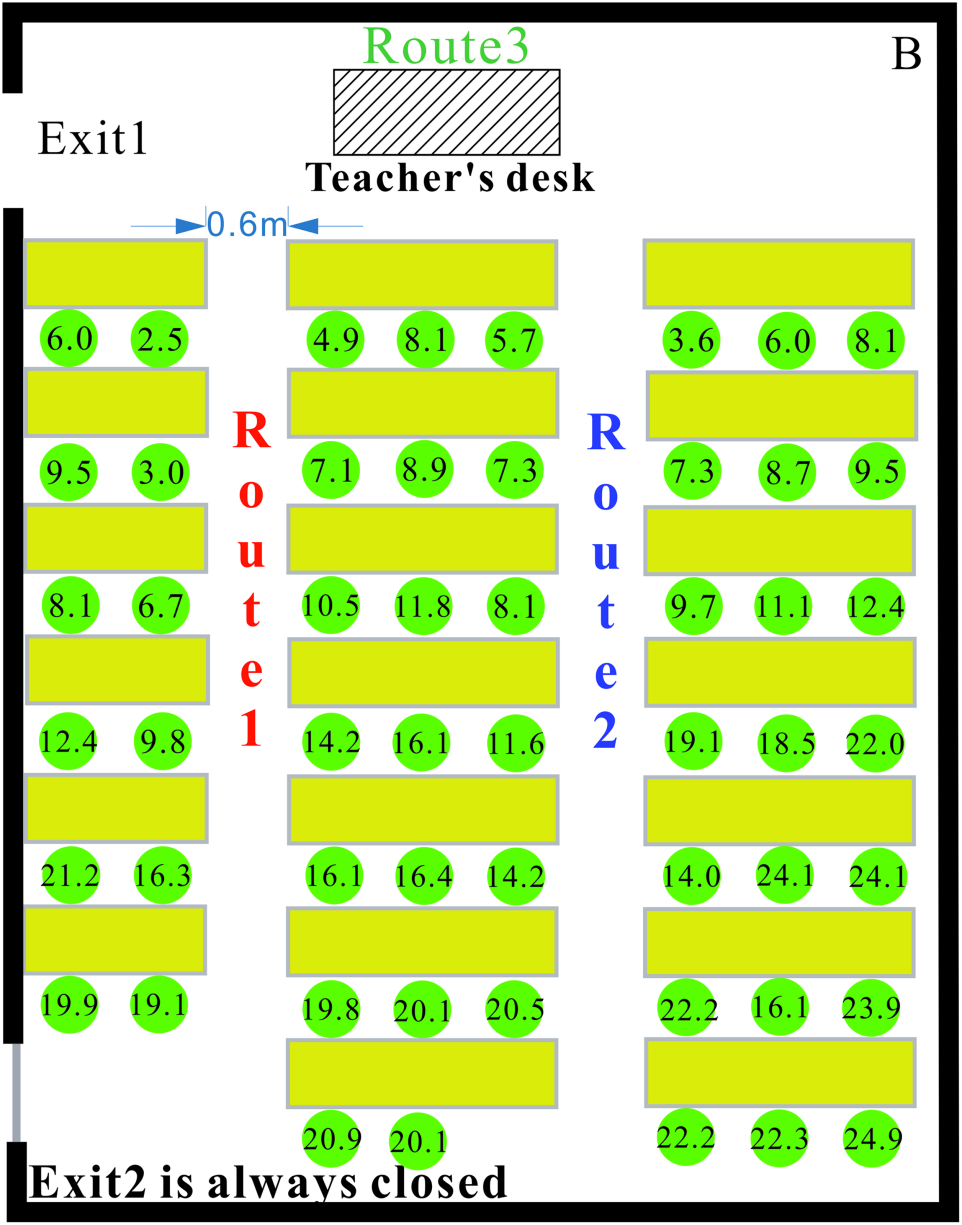
The evacuation time of each student in the Tongliao earthquake.

As shown in Fig 14, Exit 2 is always closed during the entire evacuation process. No students opened the back door to evacuate from the classroom. Students are not normally allowed to leave the classroom through the back door. Thus, the students do not take the initiative to open the back door when evacuating during the earthquake. Even the students located near the back door line up at the back of the crowd. They cannot escape the classroom until the students in front of them have evacuated. The entire evacuation process requires 24.93 s (Fig 15).

Based on the above results, we can infer that the student’s route choice is affected by the following factors. Eq (6) proposes the relationship among the parameters that are considered by a student when making their evacuation choice:

$$P\left(r,e\right)=f\left(D_{e}^{\text{min}},M_{r},{\bar{W}}_{r},n_{r},O_{r}\right),r\in R,e\in E.$$ (6)

**Fig 16:**
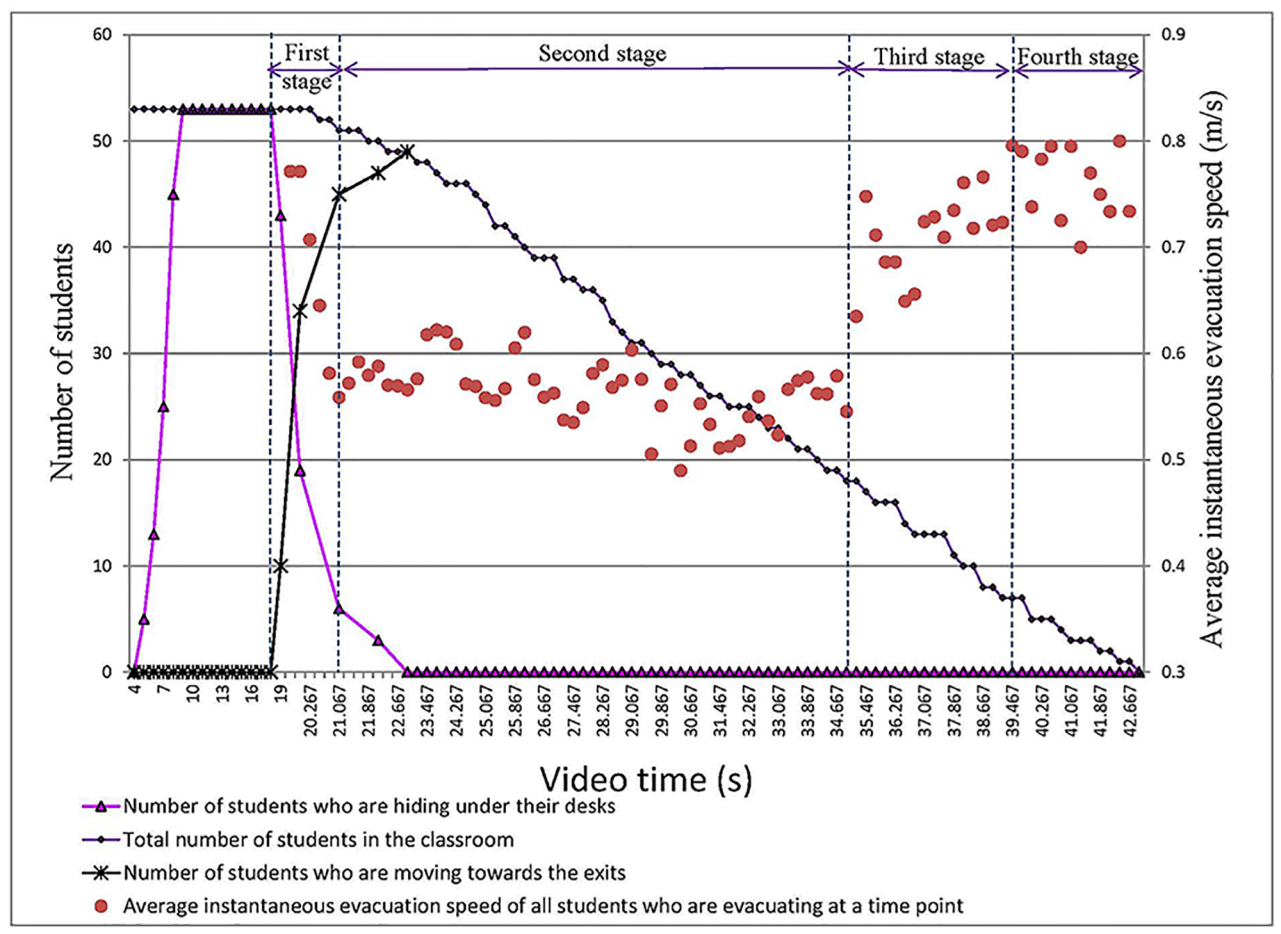
The four stages of the entire evacuation process in the No. 2 Middle School of Ganqika.

As shown in Fig 16, the origin of the coordinate system indicates the time when the earthquake occurs. The students first choose to hide under their desks when the earthquake occurs (4.00 s). At 9.00 s, all 53 students are under their desks.

To better understand the effects of classroom layout on evacuation speed, the evacuation is divided into four stages (Table 9).

**Table 9:** The number of students evacuated through routes 1–3, the front and back door during the four evacuation stages in the No. 2 Middle School of Ganqika.

	Number of evacuated students	First stage	Second stage	Third stage	Fourth stage
Route 1	28	1	19	4	4
Route 2	23	2	15	5	1
Route 3	2	0	2	0	0
Front door	53	2	33	11	7
Back door	/	/	/	/	/

From 18.00–21.07 s, the students stop hiding and begin evacuating. At 19.00 s, 10 students have already begun evacuating, and their average instantaneous speed is 0.77 m/s. Because the crowd density along routes is low, the 10 students can evacuate relatively quickly. At this time, the other students are still hiding, and they are excluded from the calculation of the average instantaneous speed. As time progresses, an increasing number of students begin to evacuate. At 21.07 s, 45 students have already begun to evacuate. Due to the large number of students and the narrowness of the evacuation routes, an increasing number of students form congestion in the routes. The students who comprise this congestion have been included in the calculation of the average instantaneous speed, resulting in a decrease in this speed.

From 21.07–34.93 s: At 22.93 s, all students have begun evacuating, and four students have escaped the classroom. During this stage, the total number of students in the classroom decreases from 51 to 18. Two of the evacuation routes are always crowded with students, whose instantaneous speeds are very low (approximately 0.50 m/s). Then, these students are included in the calculation of the average instantaneous speed. During this stage, the number of students along the routes is approximately constant. The students in the back can use the route only after the students in the front have left. During this stage, the average instantaneous speed is low, between 0.49 and 0.62 m/s.

From 34.93–39.47 s: During this stage, the number of students causing congestion along the routes gradually decreases. That is, the number of students who reduce the instantaneous speed decreases. Therefore, the average instantaneous evacuation speed increases over time from 0.55 to 0.80 m/s.

From 39.47–42.93 s: During this stage, the total number of students in the classroom decreases from 7 to 0, and the remaining students are not crowded along routes. Therefore, the average instantaneous evacuation speed is relatively steady between 0.70 and 0.80 m/s.

### Emergency exit choices

In this paper, we analyze the students’ emergency exit choices in Class 3 of Grade 7 in the Middle School of Tangshan Yucai (May 28, 2012 Tangshan earthquake). In this example, the position of each student is noted every three frames from the moment the student leaves his seat.

**Fig 17:**
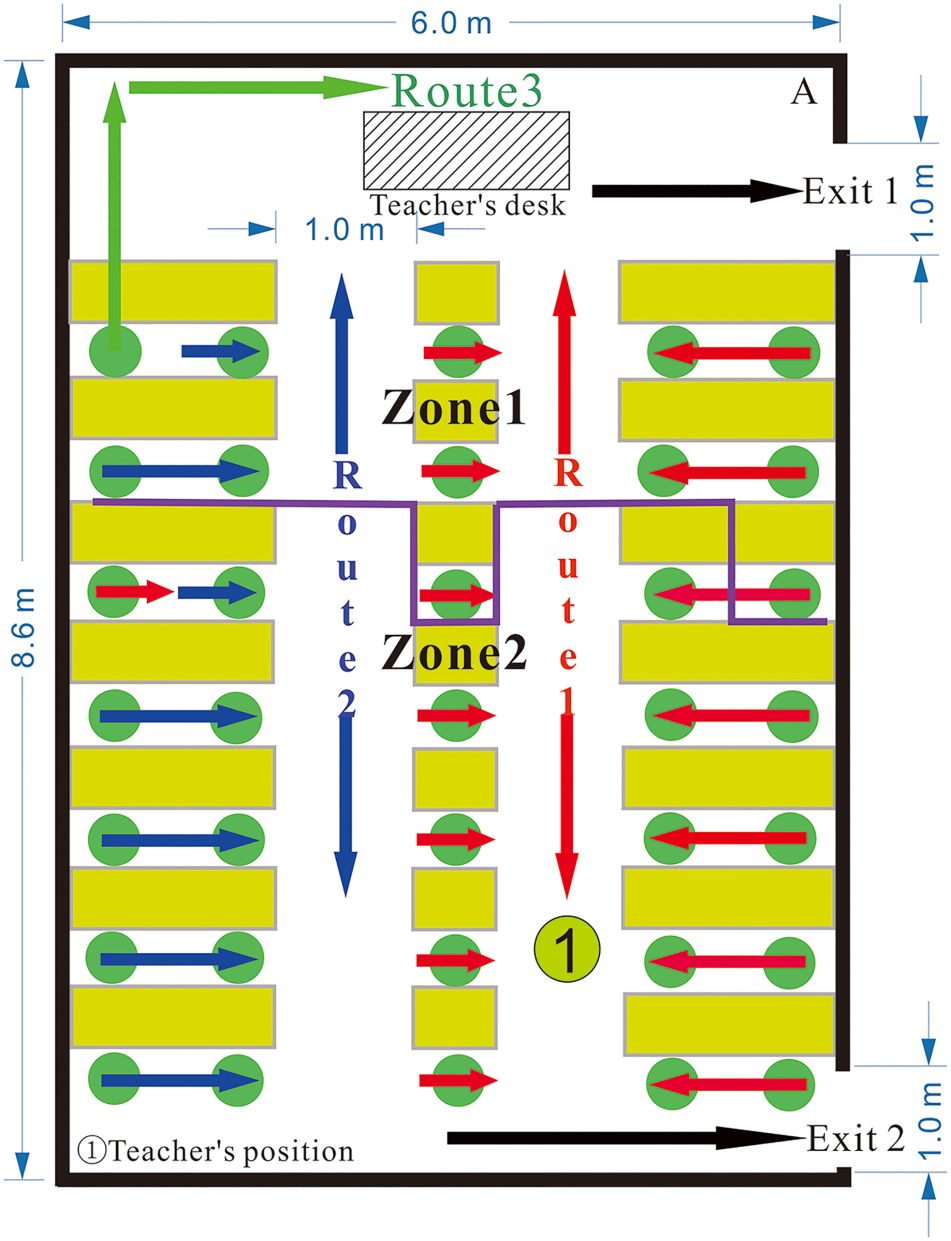
The layout and Evacuation trajectories of Class 3 in the Middle School of Tangshan Yucai.

**Fig 18:**
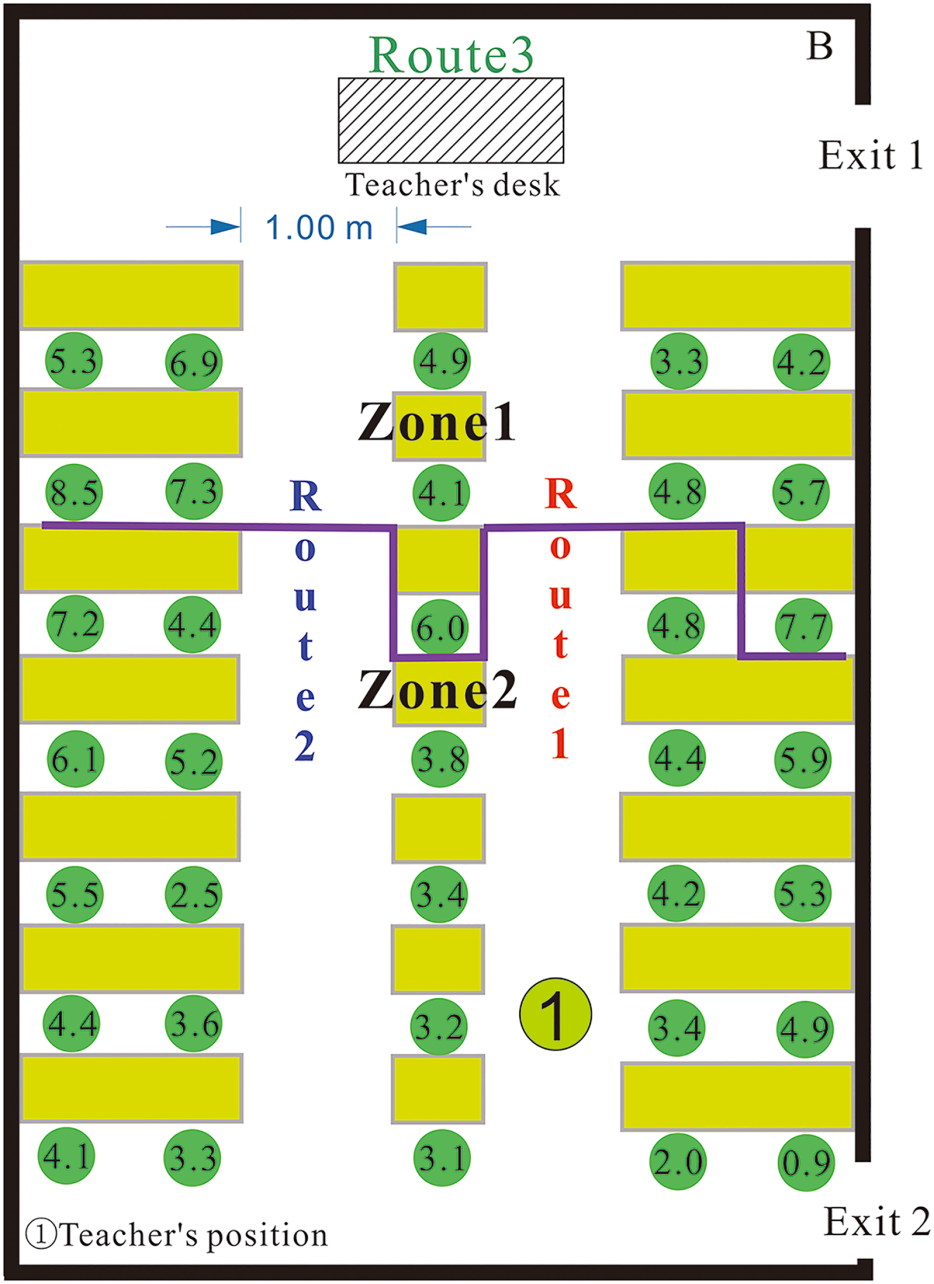
The evacuation time of each student in the Tangshan earthquake.

The classroom layout and evacuation trajectories are shown in Fig 17. There are 30 students, two emergency exits and three evacuation routes in the classroom. According to Fig 17, 12 students in Zone 1 evacuate from the classroom through Exit 1. Because the teacher is located at Exit 2 to guide students during evacuation, 18 students in Zone 2 evacuate from the classroom through Exit 2. Three students near Exit 1 choose to evacuate from the classroom through Exit 2. Thus, the evacuation guidance provided by the teacher influences the evacuation decisions of the students. Based on proximity, the students in the middle column of the classroom evacuate using Route 1. The time required for evacuation is 8.50 s, which is considerably less than the evacuation time noted for the No. 2 Middle School of Ganqika (23.93 s Fig 18).

Based on the above research, we can conclude that the student’s exit choice is affected by the following factors. Eq (7) proposes the relationship among the parameters that are considered by a student when making their evacuation choice:

$$P\left(e\right)=f\left(O_{e},D_{e}^{\text{min}},M_{e},n_{e}\right),e\in E.$$ (7)

Eqs (6) and (7) have been proposed by previous studies [32], but some changes have been made on these equations. For example, some of the unverified factors in this article are deleted. The factors left in this article have been verified by the contrast evacuation experiment in the No. 2 Middle School of Ganqika and the Middle School of Tangshan Yucai.

**Fig 19:**
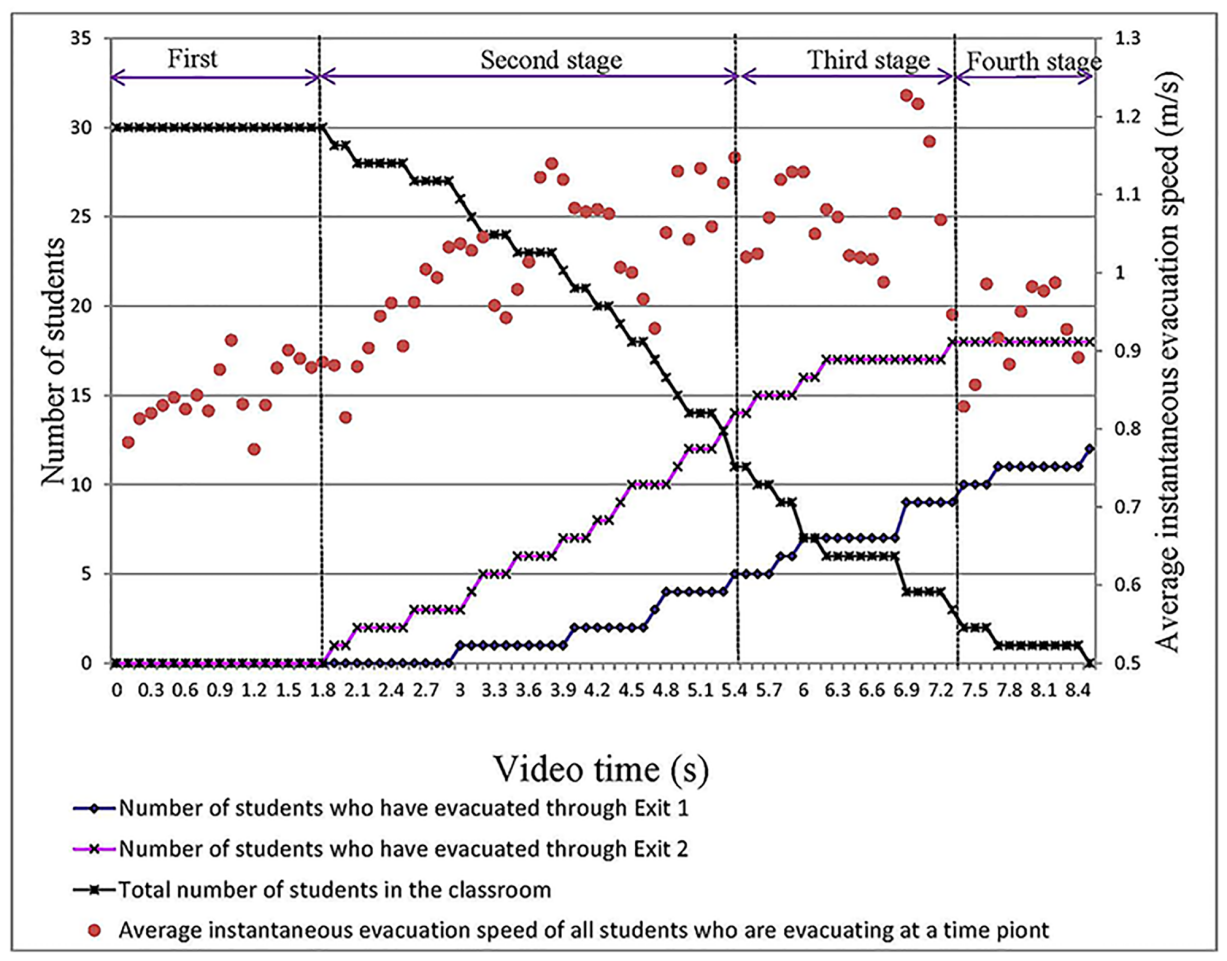
The four stages of the entire evacuation process in the Middle School of Tangshan Yucai.

In Fig 19, the origin of the coordinate system indicates the time when the students begin evacuating. At 1.90 s, the first student has evacuated from the classroom through Exit 2. At 3.00 s, the first student evacuated from the classroom through Exit 1.

To understand the impact of classroom layout on evacuation speed, we again divide the evacuation process into four stages. In contrast with the classroom in the No. 2 Middle School of Ganqika, both Exits 1 and 2 of the classroom can be used for the evacuation, and the evacuation routes are relatively wide (Table 10 shows a detailed comparison of the two classrooms in the No. 2 Middle School of Ganqika and the Tangshan Yucai Middle School).

**Table 10:** A detailed comparison of two classrooms in the No. 2 Middle School of Ganqika and the Tangshan Yucai Middle School.

	No. 2 Middle School of Ganqika				Middle School of Tangshan Yucai			
	Number of doors	Number of routes	Width of routes	Number of students	Number of doors	Number of routes	Width of routes	Number of students
Layout of classroom	1	3	0.6 m	53	2	3	1.0 m	30
	Duration (s)	Average evacuation speed (m/s)	Evacuation speed range (m/s)	Number of evacuated students	Duration (s)	Average evacuation speed (m/s)	Evacuation speed range (m/s)	Number of evacuated students
First stage	1.33	0.70	0.56–0.77	2	1.90	0.85	0.77–0.91	1
Second stage	13.87	0.56	0.49–0.62	35	3.50	1.02	0.82–1.15	19
Third stage	4.53	0.72	0.64–0.80	46	1.90	1.08	0.99–1.23	27
Fourth stage	3.47	0.76	0.70–0.80	53	1.20	0.91	0.83–0.99	30
Entire evacuation	24.93	0.63	0.49–0.80	53	8.50	0.98	0.77–1.23	30

From 0.00–1.90 s: The students stop hiding and begin evacuating. As the students transition from hiding to standing and evacuating along the two routes, the average instantaneous speed of the students increases slowly. At this time, other students are still hiding, and they are excluded from the calculation of the average instantaneous evacuation speed. As time progresses, a greater number of students begin evacuating using the routes. During this stage, the average instantaneous evacuation speed is between 0.77–0.91 m/s.

From 1.90–5.40 s: During this stage, the total number of students in the classroom decreases from 29 to 11. Due to the relatively small number of students and wide evacuation routes (approximately 1.0 m) and two exits, the students do not cause congestion along the routes, and the instantaneous speed of students is less affected by the environment and other people. These students have a high instantaneous speed s up to 1.14 m/s. As the number of students in the routes increases, the average instantaneous speed also increases. This quantity ranges from 0.82 to 1.14 m/s.

From 5.40–7.30 s: During this stage, the total number of students in the classroom decreases from 11 to 3, and the number of students along routes gradually declines. That is, the number of students who contribute to increasing the instantaneous evacuation speed decreases. Therefore, the average instantaneous evacuation speed decreases and ranges from 1.23 to 0.99 m/s.

From 7.30–8.50 s: The remaining two students in the classroom are near the front door. During this stage, these two students are evacuating from the classroom to the hallway of the building. Therefore, the average instantaneous evacuation speed is lower than that of the classroom. The speed, however, is relatively stable and ranges from 0.83 to 0.99 m/s.

Based on Table 10, the average evacuation speeds in the first stage are 0.70 m/s and 0.85 m/s for the No. 2 Middle School of Ganqika and Tangshan Yucai Middle School, respectively. In the fourth stage, the average evacuation speeds for the No. 2 Middle School of Ganqika and Tangshan Yucai Middle School are 0.76 m/s and 0.91 m/s, respectively. The above analysis shows that the average speed difference is low in the first and fourth stages between the two schools. The main speed difference is reflected in the second and third stages.

Due to the narrowness of evacuation routes, the large number of students and the smaller number of exits, the average evacuation speeds noted in the second and third stages are slower than those of the first and fourth stages in the No. 2 Middle School of Ganqika. In contrast, the average evacuation speeds in the second and third stages are quicker than those of the first and fourth stages in the Tangshan Yucai Middle School (Table 11).

**Table 11:** The number of students evacuated through routes 1–3 and Exits 1 and 2 during the four evacuation stages in the Tangshan Yucai Middle School.

	Number of evacuated students	First stage	Second stage	Third stage	Fourth stage
Route 1	20	1	17	2	0
Route 2	9	1	7	1	0
Route 3	1	0	1	0	0
Exit 1	12	0	5	4	3
Exit 2	18	1	13	4	0

## Conclusion

Quantifying the EERB of pedestrians during an earthquake emergency is of the utmost importance when developing models. In this work, based on analysis of the earthquake evacuation data of more than one hundred individuals, we construct a complete and unitary (though not exhaustive) database that encompasses the qualitative and quantitative aspects of the EERB of pedestrians for evacuation simulation software and could be used to verify the accuracy of simulation results. The constructed database is composed of the following seven parts: individual characteristics, pre-earthquake status, pedestrian response behaviors, delay time, evacuation speed, and evacuation route and emergency exit choices.

1. The qualitative data of the individual characteristics, pre-earthquake status and pedestrian response behaviors comprise the keywords and keyword groups, which provide a foundation for further study of the codification of earthquake emergency response modes under different seismic intensities (I-IX). In this paper, the evacuation scenarios that we discuss are indoor scenarios, and evacuation and hiding under safety places are the main first protective behaviors.
2. The delay time data follow the log-normal distribution. Our analysis on delay time in earthquake conditions is the first in the literature. Then, we construct a seismic intensity-gender matrix of the average delay time. The average delay time decreases as the intensity increases.
3. The instantaneous evacuation speed data follow a log-normal distribution. Then, we construct the seismic intensity-location matrix of average evacuation speed. The average evacuation speed increases with increasing seismic intensity and decreases with increasing population density.
4. The route choices of pedestrians are primarily affected by the geometric distance between the pedestrian and the nearest emergency exit, the average width of the selected route, pedestrians’ daily habits and the passing capacity of the chosen route. To understand the effects of classroom layout on evacuation speed, the evacuation process of students is divided into four stages.
5. The emergency exit choices of students are mainly affected by their teacher’s guidance, the geometric distance between the student and the nearest emergency exit, the students’ daily habits and the number of other students between the student and the nearest emergency exit.

The constructed database could be valuable in evaluating the results of evacuation simulation software programs and may provide reliable basic data for EERB models in indoor scenarios. In future research, the number of videos used in this article and analyses on the EERB should be expanded; a study of pedestrians’ earthquake evacuation in outdoor scenes should be performed; and the earthquake emergency response modes under different seismic intensities (I-IX) should be studied.

In subsequent studies, the machine learning method will be used to construct a decision tree of earthquake emergency responses. Using the decision tree, the decision rules that can predict the emergency response decisions of individuals in future earthquakes are extracted. Based on the constructed database and decision rules, computer simulation technology can be used to further study how to optimize the spaces and configurations of classrooms and how to design earthquake emergency response procedures.

## Supporting information

S1 File Database of the selected social surveillance videotapes. (XLSX)

S2 File Appendix A. Notations.
(DOCX)
