# Supplementary material for: Developing a database for pedestrians’ earthquake emergency evacuation in indoor scenarios
Source: PLoS One. 2018 Jun 20;13(6):e0197964. doi: 10.1371/journal.pone.0197964 (PMC6010270; doi:10.1371/journal.pone.0197964)
Supplement: S2 File — Notations. (DOCX) [file pone.0197964.s002.docx]

Appendix A. Notations

| The variable quantities | Unit measure | Description |
| --- | --- | --- |
| T | s | Time |
| V | m/s | Speed |
| L | number | The step size: the number of video frames at each move step |
| N/M | number | The number of individuals |
| $\text{k}\left(j\right)$ | number | The number of time points used in calculating the instantaneous speed |
| $\vec{\text{x}}\left(\text{i}\right)$ | m | Displacement |
| $T_{j}$ | s | The duration time of the entire emergency evacuation process of an individual (j) |
| $T_{\text{z}}$ | s | The time required for all individuals to evacuate out of the room |
| Z | - | The entire evacuation process |
| $j$ | - | Individual |
| eva | - | Evacuation |
| $\Delta t^{'}\left(i\right)$ | s | The short time interval |
| $t\left(i\right)$ | s | Time point |
| $Si$ | CSIS (I- IX) | Seismic intensity |
| $Lo$ | - | Location |
| $Ac$ | - | Action (What were pedestrians doing when the earthquake occurred?) |
| $Sc$ | - | Social context |
| $Ed$ | - | The emergency drill experience |
| $D_{e}^{\min}$ | m | The minimum value of geometric distance between the student and the nearest emergency exit |
| $M_{r}$ | - | The parameter of influence concerning the link between daily habits and the use of the evacuation route |
| $\bar{W}_{r}$ | m | The average width of the selected route |
| $O_{r}$ | - | The parameter of influence about the presence of teachers in the evacuation route |
| $n_{\text{r}}$ | number | The number of students using the selected route |
| $R$ | number | The total number of routes in the room |
| $E$ | number | The total number of exits in the room |
| $O_{e}$ | - | The parameter of influence with respect to the presence of teachers at the evacuation exit |
| $M_{e}$ | - | The parameter of influence with respect to the daily habits linked to the use of the evacuation exit |
| $n_{e}$ | number | The number of persons between the student and the nearest emergency exit |
